# Evaluating short-term survivors of glioblastoma: A proposal based on SEER registry data

**DOI:** 10.1093/noajnl/vdaf036

**Published:** 2025-02-09

**Authors:** Yusuke Tomita, Yoshihiro Otani, Ryo Omae, Ryo Mizuta, Joji Ishida, Nobuyuki Hirotsune, Shota Tanaka

**Affiliations:** Department of Neurological Surgery, Okayama University Medical School, Okayama, Japan; Department of Neurosurgery and Neuroendovascular Surgery, Hiroshima City Hiroshima Citizens Hospital, Hiroshima, Japan; Department of Neurological Surgery, Okayama University Graduate School of Medicine, Dentistry and Pharmaceutical Sciences, Okayama, Japan; Department of Neurological Surgery, Okayama University Graduate School of Medicine, Dentistry and Pharmaceutical Sciences, Okayama, Japan; Department of Neurological Surgery, Okayama University Graduate School of Medicine, Dentistry and Pharmaceutical Sciences, Okayama, Japan; Department of Neurological Surgery, Okayama University Graduate School of Medicine, Dentistry and Pharmaceutical Sciences, Okayama, Japan; Department of Neurosurgery and Neuroendovascular Surgery, Hiroshima City Hiroshima Citizens Hospital, Hiroshima, Japan; Department of Neurological Surgery, Okayama University Graduate School of Medicine, Dentistry and Pharmaceutical Sciences, Okayama, Japan

**Keywords:** glioblastoma, long-term survivor, SEER, short-term survivor, United States

## Abstract

**Background:**

Glioblastomas (GBMs) are central nervous system tumors with a poor prognosis and limited treatment options. Although small subsets of GBM patients survive longer than 3 years, there is little evidence regarding the prognostic factors of GBM. Therefore, we conducted a thorough characterization of GBM in the United States.

**Methods:**

We queried the Surveillance, Epidemiology, and End Results database between 2000 and 2021 to extract age-adjusted incidence rates (AAIRs), age-adjusted mortality rates (AAMRs), and survival data for GBM. We compared trends in AAIR, AAMR, and survival time across age groups 0–14, 15–39, 40–69, and 70+ years. Also, we employed the Fine–Gray competing risk model among short-term survivors (STSs), defined as those with a survival time of 6 months or less, and long-term survivors (LTSs), defined as those with a survival time of 3 years or more.

**Results:**

This study included 60 615 incident GBM cases, 54 998 GBM-specific deaths, and 47 207 GBM patients with available survival time between 2000 and 2021. The mortality-to-incidence ratio was constant among STSs, whereas it increased with age among LTSs. Higher age and male sex were significantly associated with GBM-specific death among LTSs, whereas non-Hispanic White and less intensive treatments were associated with GBM-specific deaths among STSs. Interestingly, higher age was significantly associated with other causes of death among STSs.

**Conclusions:**

STSs partially consist of populations who died from causes other than GBM. It is important to include only GBM-specific deaths in STS groups to conduct reproducible research comparing STSs and LTSs.

**Key Points**

1. Long-term survivors have a higher risk of GBM-specific death in older age and among males.

2. Short-term survivors have a higher risk of death from other causes in older age.

3. The impacts of treatments for GBM diminish among long-term survivors.

**Importance of the Study**

Glioblastomas (GBMs) generally have poor prognosis, but small subsets of GBM patients survive longer than 3 years. Although recent studies have investigated prognostic factors using clinical and molecular data, there is no consensus on these factors. One plausible examination is that some cases involved deaths from causes other than GBM, indicating that research focused on death records is needed to examine the proportion of each cause of death among GBM patients. Here, we conduct a comprehensive characterization of GBMs in the United States using the SEER database, demonstrating that higher age groups were significantly associated with other causes of death among short-term survivors. Our data suggest that it is important to include only GBM-specific deaths among short-term survivors to conduct reproducible research comparing short-term and long-term survivors.

Glioblastoma (GBM) is a central nervous system tumor designated by the World Health Organization (WHO) as grade 4, indicating that GBMs are generally fast-growing, malignant, and associated with short-term survival.^1^ The current standard therapy for GBM is maximal safe resection followed by temozolomide, radiation therapy, and tumor treating fields therapy. Despite numerous preclinical and clinical trials,^2–4^ curative treatment has yet to be established.

There are small subsets of GBM patients who survive more than 3–5 years,^5,6^ referred to as long-term survivors (LTSs), comprising 5% of GBM patients.^7^ Several studies have suggested clinical data associated with LTSs,^8–10^ whereas a recent observational study indicated inaccuracies in using clinical data to predict estimated survival time.^11^ Despite the emphasis on evaluating molecular features,^12,13^ such examinations are not universally available. Therefore, a comprehensive analysis of GBM patients using large databases is necessary to identify clinical factors associated with LTSs.

The Surveillance, Epidemiology, and End Results (SEER) database comprehensively collects cancer vital statistics from population-based registries covering nearly 50% of the US population. This database is a valuable resource widely utilized in numerous cancer epidemiology studies.^4,14^ The large sample size of SEER allows for investigating mortality rates and conducting subgroup analyses based on baseline characteristics, particularly for rare cancers. Therefore, we will leverage SEER to quantify trends in age-adjusted incidence rates (AAIRs), age-adjusted mortality rates (AAMRs), and 5-year survival rates. Additionally, we aim to identify clinical factors associated with GBM-related prognosis that differ between LTSs and short-term survivors (STSs).

## Materials and Methods

This study utilized deidentified data from the SEER database, a publicly available cancer data set maintained by the National Cancer Institute, which captures cancer registry data for approximately 48% of the US population (https://seer.cancer.gov/data/). The data set includes demographics, primary tumor site, tumor morphology, stage at diagnosis, treatments such as radiotherapy and chemotherapy, and vital status (including date of death). This secondary analysis of deidentified data was reviewed and approved by the Okayama University ethics board.

We queried cancer statistics using SEER*Stat software version 8.4.3 (Information Management Service, Inc.) between January 1, 2000, and December 31, 2021. The study period was chosen based on the publication of the third version of the WHO Classification of Tumors of the Central Nervous System, which provides clinicopathological characteristics of malignant brain tumors including GBM. We extracted the AAIR and AAMR data for GBM (Site and Morphology-SEER Brain and CNS Recode, 1.1.2 Glioblastoma). We utilized the incidence-based mortality rate to compute AAMR, as previously reported.^14,15^ Also, all the decedents included in the AAMR data were dead attributed to GBMs. All cases were histologically confirmed. Annual population estimates (denominators) between 2000 and 2021 were obtained from SEER*Stat software, utilizing bridge-race postcensal estimates of the July 1 resident population. Race bridging is a method that ensures multiracial and single-race data collection systems are sufficiently comparable to estimate and analyze race-specific statistics (https://www.cdc.gov/nchs/nvss/bridged_race.htm).

We examined the overall trends in AAIRs and AAMRs for GBM over time, comparing these trends across 4 age groups (0–14 years, 15–39 years, 40–69 years, and 70+ years) and by sex. Average AAIRs and AAMRs in each 5-year age group were compared between LTSs and STSs, as well as by sex, except for the “0” age category which was separately classified. In this study, LTS was defined as GBM cases with a survival time of 3 years or longer, STS was defined as those with a survival time of 6 months or shorter, and intermediate-term survivor was defined as those with a survival time more than 6 months but less than 3 years.

The mortality-to-incidence ratio (MIR), an indicator of survival relative to incidence, was calculated as previously described.^14^ We compared MIRs with LTS and STS, and by sex, across all 5-year age groups. The number of GBM diagnoses and population estimates (denominators) used for AAIRs and AAMRs by sex, age groups, and race/ethnicity are detailed in Supplementary Tables 1–5.

We calculated AAIRs and AAMRs per 100 000 population using yearly population estimates standardized to the US population (https://seer.cancer.gov/stdpopulations/) for the year 2000. We analyzed trends over time by estimating the average annual percent change (AAPC) using the Joinpoint Regression Program from the National Cancer Institute. This program calculates the AAPC with a 95% confidence interval (CI) and identifies statistical differences between each regression estimate.^14^ We also examined AAIRs and AAMRs of STSs and LTSs across 5-year age groups, and analyzed the trend of MIRs of STSs and LTSs across these age groups using Spearman’s rank correlation.

To facilitate survival analysis among GBM patients, we also extracted GBM-specific survival data. Isocitrate dehydrogenase (IDH) status is a crucial parameter not only for GBM diagnosis but also as a prognostic factor.^16^ However, IDH status is included in the SEER database only for cases enrolled in 2016 and onwards. It is important to evaluate whether GBM cases with or without IDH status exhibit similar prognoses to IDH-wildtype GBM, as the presence of potentially mixed IDH-mutant GBM could affect our results.

We used Kaplan–Meier curves to estimate the cumulative incidence of deaths among GBM cases with or without IDH-mutation status. Overall survival comparisons were made between IDH-wildtype GBM and GBM cases with unknown IDH status using the log-rank test. For this analysis, we only extracted the cases enrolled after 2016, when the WHO classification for central nervous system tumor introducing IDH-mutation status was published, to eliminate the influence of the remarkable progress and changes in the diagnosis of GBM.

Among LTSs of GBM, the proportion of deaths from non-cancer causes, such as cardiovascular disease and cerebrovascular diseases, is expected to increase. Best et al. indicated that 6% of GBM patients died from causes other than the GBM, including heart diseases, pneumonia and influenza, cerebrovascular diseases, accidents, adverse effects, and infections.^17^ Therefore, in the present study, we employed Fine–Gray’s competing risk models to account for competing death events. However, as shown in Supplementary Table 6, deaths from non-cancer causes were relatively rare compared to GBM-specific deaths, so all non-GBM-specific deaths were collectively categorized as other causes of death (CODs).

The follow-up duration was defined as the period between the date of diagnosis and the date of death or date of censoring at the end of 2021. We utilized univariate and multivariate Fine–Gray’s competing risk models to examine whether age category, sex, tumor location, race/ethnicity, initiation of treatment within 21 days after diagnosis, extent of resection (gross total resection [GTR], subtotal resection [STR], or other), radiation therapy, and chemotherapy were associated with deaths among individuals with GBM. Tumor locations were categorized into cerebral cortex, cerebellum, midline, and others.^14^ The extent of resection was divided into 3 groups (GTR, STR, and others), as previously reported.^18^

Subdistribution hazard ratios (SHRs) for 3 age categories (15–39 years, 40–69 years, and 70+ years) were computed using the youngest age category (0–14 years) as the reference and were presented using a Forrest plot. We also evaluated trends for linear association.

All figures were created using GraphPad Prism (version 10.2.3). Logistic regression analysis, Fine–Gray’s competing risk models and Spearman’s rank correlation were computed using R script (version 4.2.1), while other statistical analyses were conducted using GraphPad Prism (version 10.2.3).

## Results

IDH is a critical prognostic factor for GBM,^16^ and IDH-mutation status is essential for classifying diffuse gliomas.^1^ As IDH status is included in the SEER database only for cases enrolled from 2016 onwards, we compared the survival times of GBM cases diagnosed in 2016 or later with those of IDH-wildtype GBM to evaluate the impact of potentially mixed IDH-mutant GBM in the database. In the analyzed data (*n* = 14 968), 44.7% (*n* = 6689) of patients had GBMs with unknown IDH status, while 55.3% (*n* = 8279) of patients had IDH-wildtype GBMs. The median survival time of GBMs with unknown IDH status was significantly shorter than that of IDH-wildtype GBMs (Supplementary Figure 1, median survival, 12 [95% CI: 11–12] months and 13 [95% CI: 12–13] months, *P *= .007, log-rank test). Although the observation periods for IDH-wildtype GBMs were relatively short, our data suggested that the impact of potentially mixed IDH-mutant GBM cases was insignificant. Therefore, all other analyses were conducted using the total GBM data.

Overall, the present study included 60 615 incident GBMs (females 25 398 [41.9%]; males, 35 217 [58.1%]). Among these, 51 174 patients succumbed to GBM (females 21 389 [41.8%]; males, 29 785 [58.2%]). Additionally, there were 47 207 GBM patients with available survival time (females, 19 527 [41.3%]; males, 27 681 [58.7%]) observed between 2000 and 2021. The majority of GBMs were located in the cerebrum, encompassing the frontal, temporal, parietal, and occipital lobes.

GBM patients were distributed across age groups as follows: 424 cases (0.9%) in the 0–14 years age group, 2682 cases (5.7%) in the 15–39 years age group, 30 335 cases (64.3%) in the 40–69 years age group, and 13 767 cases (29.2%) in the 70+ years age group.

We initially examined trends in AAIR and AAMR to validate their comparability with past results.^19,20^ The overall trend and trends by 4 age groups (0–14 years, 15–39 years, 40–69 years, and 70+ years) in AAIRs are depicted in Supplementary Table 7. Similarly, those in AAMRs are illustrated in Supplementary Table 8.

Over the 21-year period from 2000 to 2021, overall AAIRs and the 70+ years age group showed significant increases: AAPCs of AAIRs were 0.20 (95% CI: 0.01–0.42) overall, with specific values of 1.44 (95% CI: −1.16 to 4.41) in the 0–14 years age group, 0.85 (95% CI: −0.04 to 1.87) in the 15–39 years age group, −0.01 (95% CI: −0.21 to 0.20) in the 40–69 years age group, and 0.38 (95% CI: 0.02–0.79) in the 70+ years age group. Conversely, AAPCs of AAMRs were 0.76 (95% CI: −0.23 to 1.87) overall, with specific values of 2.02 (95% CI: −1.28 to 6.14) in the 0–14 years age group, 1.58 (95% CI: 0.08–3.32) in the 15–39 years age group, 0.60 (95% CI: −0.55 to 1.89) in the 40–69 years age group, and 0.85 (95% CI: 0.10–1.69) in the 70+ years age group.

Joinpoint regression did not identify any significant inflection points between 2000 and 2021 across age groups. These findings suggest that the increase in AAMRs can be attributed to the rise in AAIRs, consistent with previous discussions.^19,20^

The overall trends by sex for AAIRs and AAMRs are summarized in Supplementary Table 9. Overall AAIRs ranged from 2.30 to 2.58 per 100 000 in females and 3.76 to 4.20 per 100 000 in males, showing consistency over the years (AAPC: females, 0.30 [95% CI: −0.01 to 0.65]; males, 0.05 [95% CI: −0.14 to 0.27]). Similarly, overall AAMRs ranged from 0.99 to 2.25 per 100 000 in females and 1.73 to 3.74 per 100 000 in males, also demonstrating stability over time (AAPC: females, 0.78 [95% CI: −0.23 to 1.95]; males, 0.64 [95% CI: −0.26 to 1.70]). Joinpoint regression did not identify any significant inflection points between 2000 and 2018 across age groups.

The overall trend and trends by race/ethnicity for AAIRs and AAMRs are presented in Supplementary Table 10. Overall AAIRs ranged from 3.45 to 3.97 per 100 000 in NHW (non-Hispanic White), from 1.44 to 2.02 per 100 000 in non-Hispanic Black (NHB), from 1.15 to 1.95 per 100 000 in NHAPI (non-Hispanic Asian and Pacific Islander), and from 2.12 to 2.79 per 100 000 in Hispanic populations. AAIR tended to increase over the years (AAPC: NHW, 0.42 [95% CI: 0.18–0.68]; NHB, 0.56 [95% CI: −0.01 to 1.26]; NHAPI, 1.16 [95% CI: 0.37–2.20]; Hispanic, 0.51 [95% CI: 0.09–1.06]).

Overall AAMRs ranged from 1.49 to 3.59 per 100 000 in NHW, from 0.89 to 1.71 per 100 000 in NHB, from 0.40 to 1.50 per 100 000 in NHAPI, and from 0.86 to 2.40 per 100 000 in Hispanic populations. AAMR significantly increased over the years (AAPC: NHW, 1.07 [95% CI: 0.00–2.25]; NHB, 1.04 [95% CI: 0.22–2.08]; NHAPI, 1.60 [95% CI: 0.58–2.98]; Hispanic, 0.89 [95% CI: 0.04–1.99]).

On the whole, each race/ethnicity exhibited similar trends in AAIRs and AAMRs during the study period compared to the overall study population.

**Figure 1.**
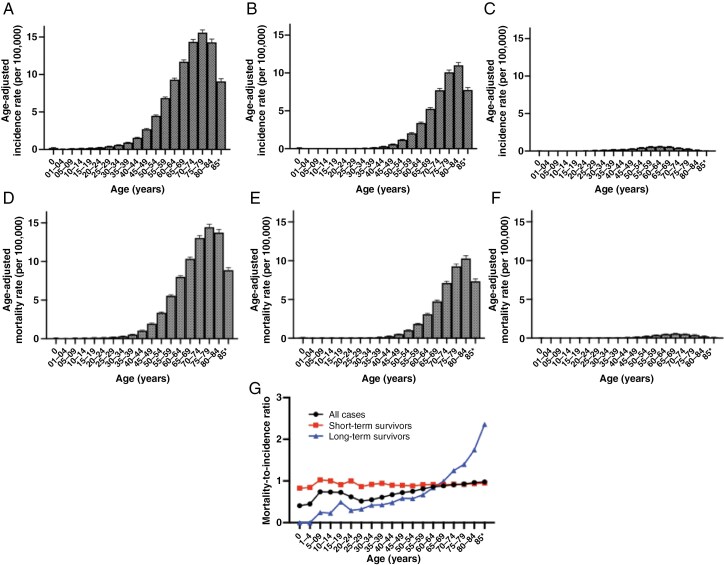
The distribution of age-adjusted incidence rate (A, all cases; B, short-time survivor; C, long-term survivor) and age-adjusted mortality rate (D, all cases; E, short-time survivor; F, long-term survivor) related to glioblastomas per 100 000 for each 5-year age group. Both age-adjusted incidence rate and age-adjusted mortality rate are summarized as the mean with standard error. (G) Mortality-to-incidence ratio using A–F data.

Figure 1 and Supplementary Table 11 illustrate differences in average AAIRs and AAMRs across 5-year age groups. Overall, average AAIRs peaked significantly in the 75- to 79-year-old group (average AAIR 15.58, 95% CI: 15.21–15.96) (Figure 1A). Similar trends were observed among STSs, peaking in the 80- to 84-year-old group (Figure 1B). In contrast, LTSs exhibited much lower AAIRs than STSs, with a mild peak observed in the 55- to 69-year-old groups (Figure 1C).

Overall, average AAMRs also peak prominently in the 75- to 79-year-old group (average AAMR 14.44, 95% CI: 14.08–14.81) (Figure 1D), showing similar trends to AAMRs among both STSs and LTSs (Figure 1E and F). The MIR calculated from AAIRs and AAMRs demonstrated a consistent increase with age among LTSs but not among STSs (Figure 1G).

Both GBM and LTSs displayed a significant association between age groups (ordinary) and MIR, with a Spearman’s rank correlation coefficient of 0.84 (*P *< .001) in all cases and 0.97 (*P *< .001) in LTSs, respectively. In contrast, as expected from the definition of STS, there was no significant correlation with age groups (Spearman’s rank correlation coefficient 0.19, *P *= .446).

Next, we extracted and analyzed SEER survival data to compare the survival time of GBM-specific death between STSs and LTSs. Table 1 presents the demographic features of the data, while Supplementary Table 6 details the CODs. Supplementary Figure 2 shows the survival curves of STSs and LTSs. At the time of data collection, the STS group comprised 16 625 GBM cases, and the LTS group comprised 4197 GBMs. The proportion of patients aged 40 years and older was significantly higher among STS (*P* < .001, multivariate logistic regression analysis). In contrast, the proportion of patients younger than 40 years of age, female, NHAPI, patients treated within 3 weeks after diagnosis, GTR, RT, and chemotherapy was significantly higher among LTS (*P* < .001, respectively, multivariate logistic regression analysis, Table 1). Supplementary Tables 12 and 13 display the results of Fine–Gray’s competing risk model assessing factors related to GBM-specific death.

**Table 1.** Comparison of Clinical Characteristics Between Short Survivors and Long Survivors

Characteristics		Total Cases (47 207)	Short-Term Survivor (16 625)	Long-Term Survivor (4197)	Univariate Logistic Regression		Multivariate Logistic Regression	
					*P*-Value	OR (95% CI)	*P*-Value	OR (95% CI)
Age group	0–14	423 (0.9%)	85 (0.5%)	84 (2.0%)	<.001	0.25 (0.19–0.34)	—	—
	15–39	2682 (5.7%)	312 (1.9%)	816 (19.4%)	<.001	0.08 (0.07–0.09)	<.001	0.45 (0.31–0.68)
	40–69	30 335 (64.3%)	8432 (50.7%)	2964 (70.6%)	<.001	0.43 (0.40–0.46)	<.001	4.33 (2.99–6.27)
	70+	13 767 (29.2%)	7796 (46.9%)	84 (2.0%)	<.001	42.99 (34.58–53.43)	<.001	27.17 (18.48–39.98)
Sex	Female	19 527 (41.4%)	7300 (43.9%)	1833 (43.7%)	.783	1.01 (0.94–1.08)	<.001	0.82 (0.76–0.90)
	Male	27 680 (58.6%)	9325 (56.1%)	2364 (56.3%)	—	—	—	—
Location	Cerebral cortex	41 549 (88.0%)	16 369 (98.5%)	4120 (98.2%)	.190	1.20 (0.93–1.55)	—	—
	Cerebellum	327 (0.7%)	133 (0.8%)	40 (1.0%)	.341	0.84 (0.59–1.19)	—	—
	Midline	356 (0.8%)	116 (0.7%)	37 (0.9%)	.224	0.79 (0.55–1.16)	—	—
Race	NHW	36 405 (77.1%)	13 171 (79.2%)	3065 (73.0%)	<.001	1.41 (1.30–1.52)	.381	1.08 (0.91–1.29)
	NHB	2612 (5.5%)	914 (5.5%)	247 (5.9%)	.328	0.93 (0.81–1.08)	—	—
	NHAPI	2334 (4.9%)	648 (3.9%)	285 (6.8%)	<.001	0.56 (0.48–0.64)	<.001	0.64 (0.50–0.82)
	Hispanic	5581 (11.8%)	1798 (10.8%)	573 (13.7%)	<.001	0.77 (0.69–0.85)	.249	0.89 (0.72–1.09)
Treat within 3 weeks	Yes	36 421 (77.2%)	11 280 (67.8%)	3531 (84.1%)	<.001	0.40 (0.36–0.43)	<.001	0.81 (0.72–0.90)
	No/unknown	10 786 (22.8%)	5345 (32.2%)	666 (15.9%)	—	—	—	—
Extent of resection	GTR	21 739 (46.1%)	5627 (33.8%)	2453 (58.4%)	<.001	0.36 (0.34–0.39)	<.001	0.39 (0.36–0.43)
	STR	16 367 (34.7%)	5518 (33.2%)	1404 (33.5%)	.755	0.99 (0.92–1.06)	—	—
Radiation therapy	Yes	35 262 (74.7%)	7902 (47.5%)	3801 (90.6%)	<.001	0.09 (0.08–0.11)	<.001	0.31 (0.27–0.36)
	No/unknown	11 945 (25.3%)	8723 (52.5%)	396 (9.4%)	—	—	—	—
Chemotherapy	Yes	30 357 (64.3%)	5697 (34.3%)	3613 (86.1%)	<.001	0.08 (0.08–0.09)	<.001	0.18 (0.16–0.20)
	No/unknown	16 850 (35.7%)	10 928 (65.7%)	584 (13.9%)	—	—	—	—

Abbreviations: GTR, gross total resection; NHAPI, non-Hispanic Asian and Pacific Islander; NHB, non-Hispanic Black; NHW, non-Hispanic White; STR, subtotal resection.

**Figure 2.**
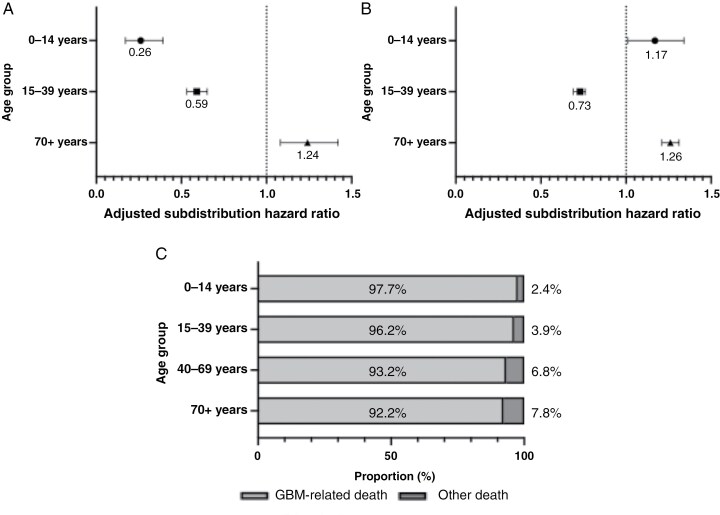
Survival analysis for glioblastoma (GBM) patients by age group. (A, B) The comparison of adjusted subdistribution hazard ratios among GBMs with longer than 3 years of survival (A) and those with less than 6 months of survival (B) by 3 age categories (0–14 years, 15–39 years, and 70+ years) using the youngest age category (40–69 years) as a referent. The subdistribution hazard ratios were calculated with Fine–Gray’s competing risk models and adjusted for age, sex, and race/ethnicity. (C) The proportion of GBM-related or GBM-unrelated death among short-term survivors. The proportion of GBM-unrelated death increased with age.

Among LTSs, compared to the 40–59 years age group, the 0–14 and 15–39 years age groups showed independent associations with lower risks of GBM-specific death, whereas the 70+ years age group were associated with higher risks of GBM-specific death (Figure 2A and Supplementary Table 13, SHR, 0–14 years age group, 0.25 [95% CI: 0.16–0.37]; 15–39 years age groups, 0.58 [95% CI: 0.52–0.64]; 70+ years age groups, 1.21 [95% CI: 1.06–1.39]). The log-rank trend test adjusted with sex showed statistically significant (*P* < .001, log-rank trend test), indicating that the older age groups were significantly associated with higher risks of GBM-specific death. In contrast, among STSs, the 0–14 and 70+ years age groups exhibited similar risks of GBM-specific death compared to the 40–69 years age group (Figure 2B and Supplementary Table 13, SHR, 0–14 years age group, 1.18 [95% CI: 0.98–1.44]; 15–39 years age groups, 1.25 [95% CI: 1.12–1.40]; 70+ years age groups, 0.99 [95% CI: 0.96–1.02]). The log-rank trend test adjusted with sex showed statistically significant (*P* < .001, log-rank trend test), suggesting that the risks of GBM-specific death were consistent regardless of age. Instead, among STS, the older age groups showed independent associations with deaths from other causes (SHR, 0–14 years age groups, 0.24 [95% CI: 0.06–0.95]; 15–39 years age groups, 0.47 [95% CI: 0.47–1.21]; 70+ years age groups, 1.21 [95% CI: 1.08–1.36]).

As for the background factors other than age, male sex was significantly associated with GBM-specific death among LTS and intermediate-term survivor but not among STS (Supplementary Tables 12 and 13). Among NHW, STSs with GBM were significantly associated with a higher risk of GBM-specific death and lower risk of other CODs compared to other races/ethnicities.

Regarding treatment, GTR, radiation therapy, and chemotherapy were significantly associated with a lower risk of GBM-specific deaths among STSs (SHR, GTR, 0.93 [95% CI: 0.90–0.96]; radiation therapy, 0.68 [95% CI: 0.66–0.71]; chemotherapy, 0.80 [0.78–0.83]), but not among LTSs (SHR, GTR, 1.09 [95% CI: 0.98–1.21]; radiation therapy, 1.10 [95% CI: 0.94–1.29]; chemotherapy, 1.13 [0.99–1.29]).

From the results of Fine–Gray’s competing risk model, we further focused on the CODs. We evaluated the proportions of GBM-specific deaths and other CODs, finding that the proportions of other CODs significantly increased with age (Figure 2C–F, *P *= .002, Fisher’s exact test). To explore the potential effects of other CODs on the survival time, we conducted multivariate Fine–Gray’s competing risk model analyses again with the data in which the cases with other CODs were enrolled as “dead” rather than “censored” (Supplementary Table 14). Although the change of SHRs was relatively small, SHR of each character increased or decreased. Of note, male sex was significantly associated with GBM-specific death in STS by handling cases with other CODs as “dead” (SHR, 1.03 [95% CI: 101–1.06], *P* = .012).

According to the survival analysis, female sex was associated with a lower risk of GBM-specific death among LTSs. To validate this finding, we calculated rate ratios using AAMR for GBM-specific death by sex and age groups. Interestingly, AAMR in females was significantly lower than that in males among both STSs and LTSs (Supplementary Table 15). Similarly, we calculated rate ratios using AAIR filtered for GBM-specific death by sex and age groups. Like AAMR, AAIR in females was significantly lower than that in males among both STSs and LTSs (Supplementary Table 16). Therefore, females exhibit a lower number and risk of GBM-specific death compared to males, irrespective of whether they are STSs or LTSs.

## Discussion

In this epidemiological study, we comprehensively described AAIRs, AAMRs, and survival trends in GBM over a 21-year period. Average AAIRs and AAMRs exhibited a pronounced peak in the 70–79 years age groups for both males and females. The MIR increased with age among LTSs, while it remained relatively stable across age groups among STSs. Age group, sex, race/ethnicity, and chemotherapy were significantly associated with GBM-specific death among LTSs, whereas age group was associated with death from other causes among STSs. As age increased, the proportion of death from other causes significantly rose among STSs. Females had a significantly lower risk of GBM-specific death compared to males. Our findings underscore the importance of caution when interpreting clinical research that compares STSs and LTSs due to their heterogeneous clinical profiles.

The competing risk analysis revealed that the adjusted SHR for GBM-specific death exponentially increased with age among LTSs. This finding aligns with the higher MIR observed in the elderly population among LTSs. In contrast, the adjusted SHR for other CODs was significantly lower in the 0–14 years and 15–39 years age groups and higher in the 70+ years age group compared to the 40–69 years age group among STSs. This underscores the importance of considering competing events such as cardiovascular deaths when evaluating mortality among STSs. This observation is supported by past SEER studies indicating a significant association between patients older than 60 years and other CODs, such as heart diseases, pneumonia and influenza, cerebrovascular diseases, accidents, adverse effects, and infections.^17^ Given the age-related increase in cardiovascular disease, it is plausible that deaths from cardiovascular causes tend to rise in older age groups.^21^ However, it is noted that the GBM cases younger than 60 years old, usually not assumed to be dead with aging-related disease, also have certain risks of deaths with other CODs. Specifically, in STSs, 7.2% of total cases and 5.9% of cases younger than 60 years old were attributed to other CODs (Supplementary Table 6), highlighting a major limitation when comparing the clinical and molecular characteristics of STSs with those of LTSs. The STS group may include individuals with other CODs who survived longer than the conventional upper limit for STS, potentially introducing noise into the population. Indeed, recent comprehensive molecular analyses of LTSs and STSs based on the Cancer Genome Atlas Program (TCGA) and Chinese Glioma Genome Atlas (CGGA) have reported varied results across studies.^22–24^ Our study suggests that 1 contributing factor to this variability is the lack of detailed COD records in TCGA and CGGA data sets, hindering reproducible analysis of differentially expressed genes. Therefore, focusing exclusively on GBM-specific deaths exclusively in STS groups is crucial for identifying reproducible and reliable prognostic factors.

Among the total GBM cases, the MIR was similar between males and females, though AAIRs and AAMRs were much higher in males than in females. Interestingly, when analyzing only the GBM-specific deaths among STSs or LTSs, the MIR in females was lower than that in males among LTSs, regardless of age groups (Supplementary Table 14). This finding suggests that sexual disparity may become more prominent among long-surviving GBM patients. A plausible explanation is that sexual differences, such as those in the immune system or hormonal factors, could be related to the risk of death, at least for LTSs. Gene expression microarray profiles of GBM tissues indicated that T cell and myeloid lineage–associated genes were enriched in LTSs.^25^ Also, Baylk et al. conducted preclinical research describing the sexual dimorphism of myeloid-derived suppressor cells using murine GBM models.^26^ On the other hand, Najem et al. recently conducted high-dimensional fluorescence multiplex staining spatial analysis of the tumor microenvironment, showing that CD8+ T cells and microglia may help determine short-term versus long-term survival.^27^ Regarding hormonal differences, previously reported SEER research has discussed the role of sex hormones in the development of GBM.^28^ Moreover, Fariña-Jerónimo et al. showed a significant negative correlation between androgen receptor activity and GBM survival.^29^ Although the statistical power might be limited among GBM cases, sexual disparity should be more comprehensively discussed in future research.

Among STSs, our data showed that NHW was significantly associated with higher risks for GBM-specific death than other races/ethnicities, including NHB, NHAPI, and Hispanic. This result is consistent with a recently reported Central Brain Tumor Registry of the US-based study.^20^ On the other hand, STS data demonstrated that NHW was significantly associated with a lower risk for other CODs compared to other races/ethnicities. One plausible explanation includes racial and socioeconomic disparities. Ramapriyan et al. conducted SEER research to examine county-level racial and socioeconomic characteristics among GBM patients, indicating that counties with a higher percentage of Black patients have lower rates of surgery and adjuvant therapy for GBM.^30^ In contrast, 2 other studies involving glioma patients reported a very limited impact of socioeconomic status on survival,^31,32^ suggesting that differences in cancer type, countries, and adjusted comorbidities may have led to different results. Therefore, it is possible that socioeconomic status may have affected the results in this study as well, but further studies are required to validate our finding.

Our competing risk analysis detected that GTR, radiation, and chemotherapy were significantly associated with a lower risk of GBM-specific deaths among STSs and intermediate-term survivors. These findings are comparable with previously conducted clinical research.^33,34^ Interestingly, our data demonstrated that GTR, radiation, and chemotherapy were not associated with GBM-specific deaths among LTSs. A plausible examination for this finding is that the effect of these treatments diminishes over time and that the adverse events of these treatments counterbalance the survival benefits. The former reason is supported by the fact that each treatment is generally conducted and completed in the first few months after diagnosis, suggesting that these treatments have only a limited impact on survival time beyond 3 years. The latter explanation might be illustrated by research examining the long-term use of temozolomide, although the SEER database does not include data on temozolomide use. The GEINO-14 trial compared the efficacy of continuing adjuvant temozolomide beyond 6 cycles with that of 6-cycle use of temozolomide. It found that continuing temozolomide beyond 6 cycles did not prolong survival but increased toxicities such as lymphopenia, thrombocytopenia, and nausea and vomiting compared with 6-cycle use of temozolomide.^35^ Also, previous radiation and chemotherapy might cause secondary neoplasms among LTSs of GBM,^36^ which is a specific problem among LTSs undergoing treatment. Preclinical and clinical research should be conducted to establish novel treatment strategies with sustainable antitumor effects for longer than 3 years.

Kawauchi et al. recently showed that surgical interventions within 3 weeks from onset are associated with prolonged survival in GBM patients, possibly due to smaller tumor volumes and better postoperative performance status.^37^ However, multivariate Fine–Gray’s analysis in this study indicated that initiating treatment within 3 weeks was associated with a higher risk of GBM-specific death among STSs, which contrasts with past reports. Also, as shown in Table 1, the proportion of patients receiving radiation and chemotherapy was significantly lower among STSs than among LTSs. A possible explanation for the discrepancy is the presence of early progression cases in the SEER database. Early progression, defined as early tumor growth validated at pre-radiotherapy MRI, is associated with poor survival.^38^ Cases with early progression are frequently excluded from the standard of care or clinical trials due to declining performance status. Interestingly, if we extracted only STS cases that received both radiation and chemotherapy, patients treated within 21 days had a significantly lower risk of death than those treated after 21 days (hazard ratio 0.88 [95% CI: 0.80–0.97], *P *= .008, log-rank test). Further studies are required to validate this finding, but it might be important to attempt completion of the standard treatment even in early progressive cases.

The strength of this study includes the use of up-to-date data on GBM incidence, mortality, and survival from 2000 to 2021, drawn from a large nationwide registry data covering approximately one-third of the US population. This broad coverage allows for generalization of the present observations, and reduces the likelihood of bias compared to conventional observation studies or single-center registries. The large sample size also enabled us to conduct several subgroup analyses to examine detailed incidence and mortality trends in GBM. By using competing risk models, we also examined in detail the impact of other competing CODs on the mortality of GBM patients, particularly among STSs.

However, this study has several limitations. First, it did not account for important confounding factors such as family, functional status, comorbidities, and detailed treatment-related information (eg, specifics of surgery or chemotherapy). Despite conducting several subgroup analyses, the effects of residual confounding should be considered when interpreting the results. Second, the study was conducted in the United States and primarily included NHW individuals, who accounted for more than 70% of the total study population. This limits the generalizability of our findings to other populations in different countries. Third, the study investigated average trends in AAIRs and AAMRs and survival in the population diagnosed with GBM. As discussed, the GBMs in this study include a heterogeneous group of tumors because the SEER database does not yet include sufficient molecular information (eg, IDH status, histone mutation, MGMT promoter methylation status). The SEER database would be more informative if it included molecular and methylome information of tumors. Fourth, the SEER database did not include all the well-known prognostic factors associated with GBM survival (eg, Karnofsky performance status, volumetric data, and numerical value for extent of resection). Especially, poor performance status is one of the important prognostic factors that could influence the physicians’ and patients’ decision-making process.^39^ The data potentially have a bias that STS had enrolled more GBM cases with poor performance status than LTS, for which STS cases underwent less intensive treatment. Future revision of the SEER database would resolve this problem. Finally, the results of this study should be interpreted with caution because factors like lead-time bias can influence analyses based on incidence-based mortality.

## Conclusions

This study highlights the need for caution when evaluating the clinical characteristics of STSs of GBM, as this group may include subsets with other CODs, especially among elderly patients. Additionally, the impact of standard treatments appears to diminish among LTSs, suggesting an urgent need to establish novel treatment strategies.

## Supplementary Material

Supplementary material is available online at *Neuro-Oncology Advances* (https://academic.oup.com/noa).

vdaf036_suppl_Supplementary_Figure_S1

vdaf036_suppl_Supplementary_Figure_S2

vdaf036_suppl_Supplementary_Materials

vdaf036_suppl_Supplementary_Table_S1

vdaf036_suppl_Supplementary_Table_S2

vdaf036_suppl_Supplementary_Table_S3

vdaf036_suppl_Supplementary_Table_S4

vdaf036_suppl_Supplementary_Table_S5

vdaf036_suppl_Supplementary_Table_S6

vdaf036_suppl_Supplementary_Table_S7

vdaf036_suppl_Supplementary_Table_S8

vdaf036_suppl_Supplementary_Table_S9

vdaf036_suppl_Supplementary_Table_S10

vdaf036_suppl_Supplementary_Table_S11

vdaf036_suppl_Supplementary_Table_S12

vdaf036_suppl_Supplementary_Table_S13

vdaf036_suppl_Supplementary_Table_S14

## Data Availability

This study utilized deidentified data from the SEER database that is publicly available.
