# Supplementary figures and images for: Evaluating short-term survivors of glioblastoma: A proposal based on SEER registry data
Source: Neurooncol Adv. 2025 Feb 9;7(1):vdaf036. doi: 10.1093/noajnl/vdaf036 (PMC12080546; doi:10.1093/noajnl/vdaf036)

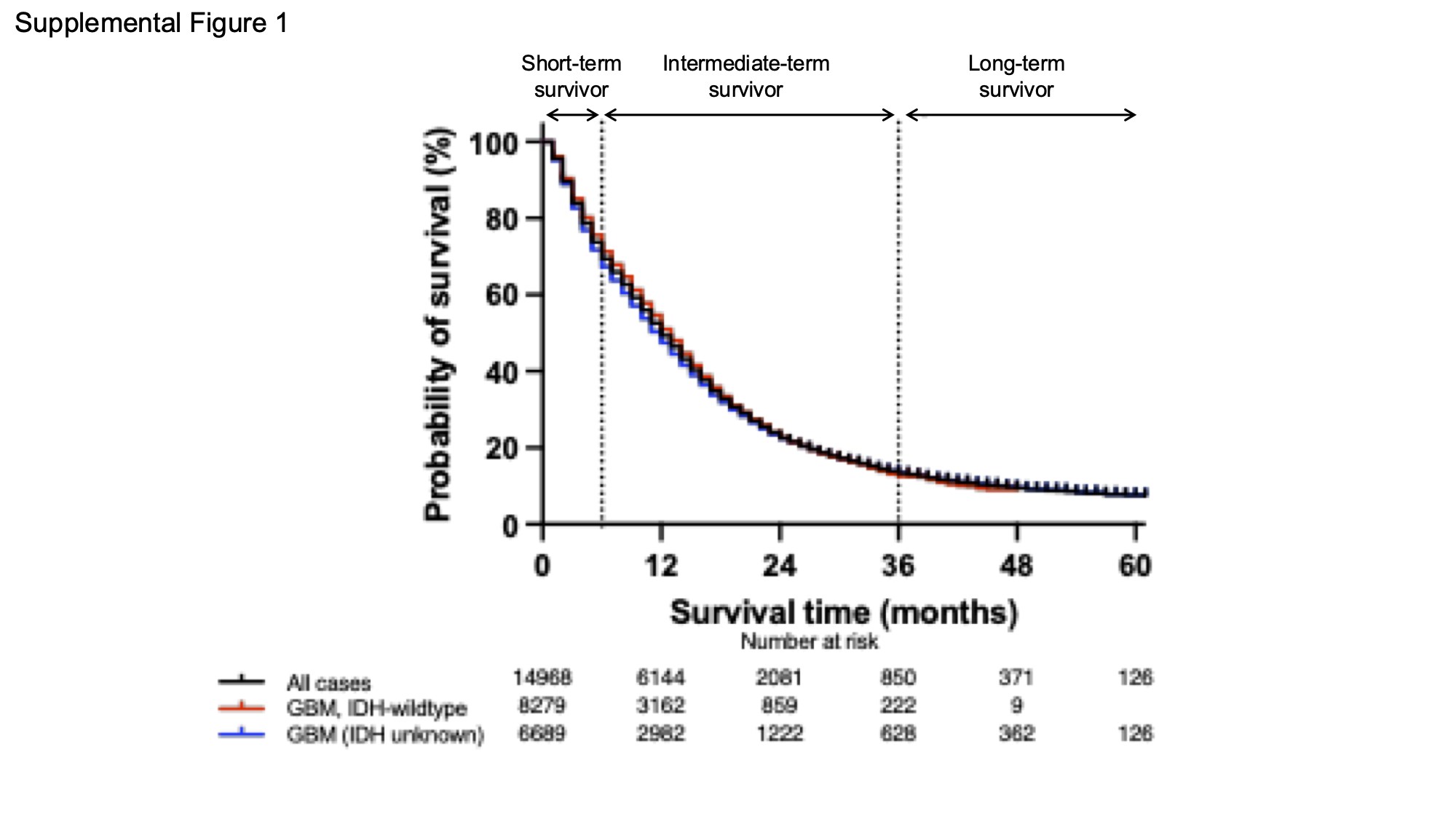

Supplement: vdaf036_suppl_Supplementary_Figure_S1 [file vdaf036_suppl_supplementary_figure_s1.jpeg]

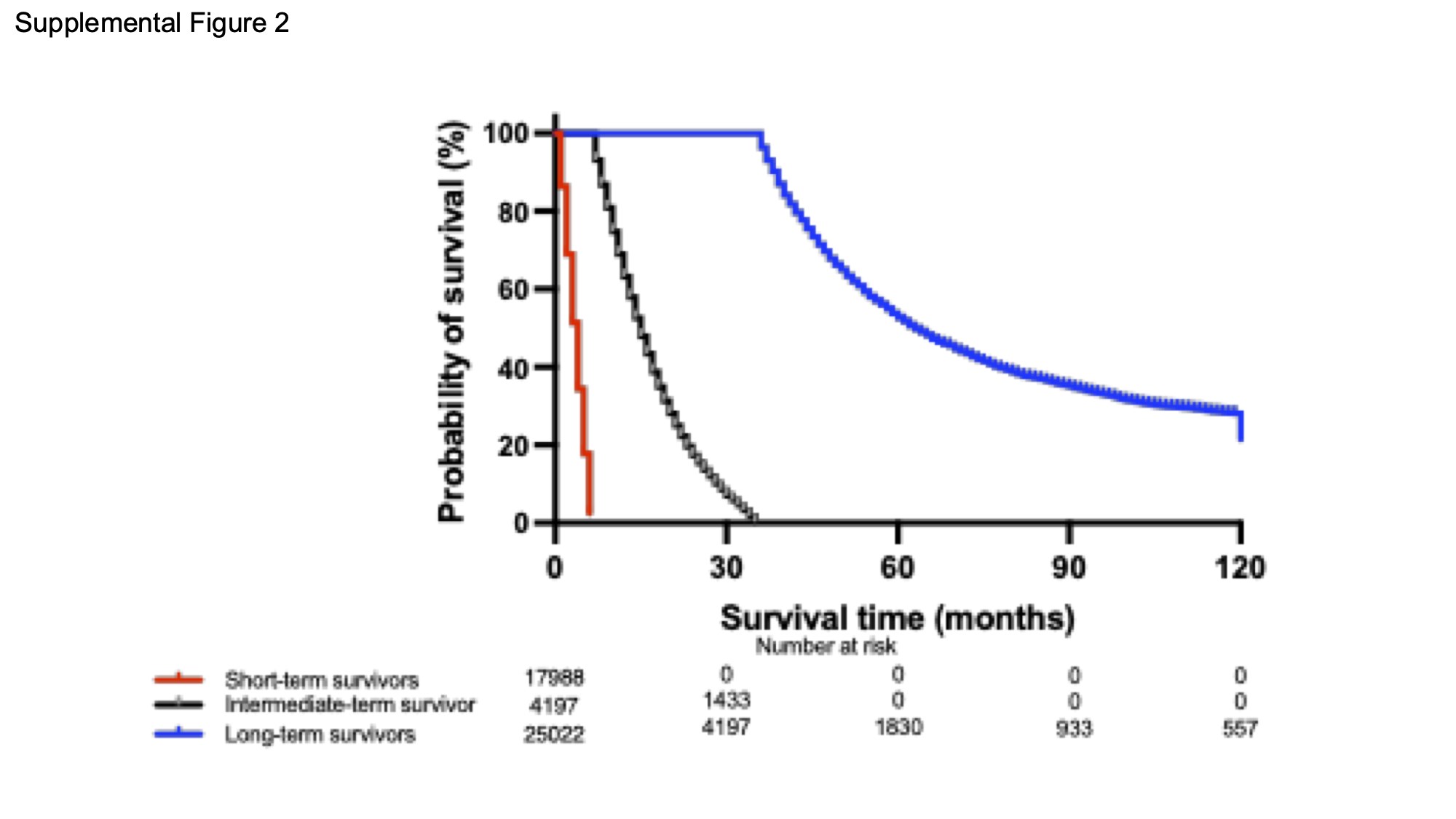

Supplement: vdaf036_suppl_Supplementary_Figure_S2 [file vdaf036_suppl_supplementary_figure_s2.jpeg]
