# Supplementary material for: Evaluating short-term survivors of glioblastoma: A proposal based on SEER registry data
Source: Neurooncol Adv. 2025 Feb 9;7(1):vdaf036. doi: 10.1093/noajnl/vdaf036 (PMC12080546; doi:10.1093/noajnl/vdaf036)
Supplement: vdaf036_suppl_Supplementary_Materials [file vdaf036_suppl_supplementary_materials.docx]

**Supplemental figure legends**

**Supplemental figure 1.** Survival analysis in glioblastomas. Survival curves over 5 years from diagnosis related to glioblastoma by IDH status: all cases, glioblastoma with unknown IDH status, and IDH-wildtype glioblastoma in 2016 and later. IDH-unknown GBMs had significantly shorter survival time than IDH-wildtype GBMs.

**Supplemental figure 2.** Survival analysis in glioblastomas with short-term survivors, intermediate survivors, and long-term survivors. Short-term survivors were defined as deceased cases within 6 months, whereas intermediate- and long-term survivors were defined as all cases including both deceased, alive, and censored cases.

**Supplemental Table legends**

**Supplemental Table 1.** Trends in the number of patients with glioblastoma and estimated population by age groups.

**Supplemental Table 2.** Trends in the number of decedents from glioblastoma and estimated population by age groups.

**Supplemental Table 3.** Trends in the number of patients and decedents from glioblastoma and estimated population by sex.

**Supplemental Table 4.** Trends in the number of patients and decedents from glioblastoma and estimated population by race/ethnicity.

**Supplemental Table 5.** Distribution of the number of patients and decedents from glioblastoma and estimated population.

**Supplemental Table 6.** Demographic features of survival data within short-time survivors and long-term survivors.

**Supplemental Table 7.** Trends in age-adjusted incidence of glioblastoma by age groups.

**Supplemental Table 8.** Trends in age-adjusted mortality of glioblastoma by age groups.

**Supplemental Table 9.** Trends in age-adjusted incidence and mortality of glioblastoma by sex.

**Supplemental Table 10.** Trends in age-adjusted incidence and mortality of glioblastoma by race/ethnicity.

**Supplemental Table 11.** Total annual age-adjusted incidence rates and age-adjusted mortality rates by 5-year-age group.

**Supplemental Table 12.** Multivariate Fine-Gray’s analysis for glioblastoma regarding cases with other causes of death as “dead” rather than “censored”.

**Supplemental Table 13.** Annual age-adjusted mortality rates and ratios in decedents with GBM-specific death by sex and each age subgroup.

**Supplemental Table 14.** Annual age-adjusted incidence rates and ratios in patients with GBM-specific death by sex and each age subgroup.
