# Supplementary material for: Evaluating short-term survivors of glioblastoma: A proposal based on SEER registry data
Source: Neurooncol Adv. 2025 Feb 9;7(1):vdaf036. doi: 10.1093/noajnl/vdaf036 (PMC12080546; doi:10.1093/noajnl/vdaf036)
Supplement: vdaf036_suppl_Supplementary_Table_S1 [file vdaf036_suppl_supplementary_table_s1.docx]

**Supplemental table 1. Trends in the number of patients from glioblastoma and estimated number of population by age groups**

|  | **All** | | **0-14 years of age** | | **15-39 years of age** | | **40-69 years of age** | | **70+ years of age** | |
| --- | --- | --- | --- | --- | --- | --- | --- | --- | --- | --- |
| **Year** | **Patients** | **Population** | **Patients** | **Population** | **Patients** | **Population** | **Patients** | **Population** | **Patients** | **Population** |
| 2000 | 2129 | 74952547 | 15 | 16,638,605 | 126 | 27,808,930 | 1192 | 24,359,021 | 796 | 6,145,991 |
| 2001 | 2143 | 75823654 | 16 | 16,698,155 | 121 | 27,896,701 | 1224 | 25,024,570 | 782 | 6,204,228 |
| 2002 | 2208 | 76597496 | 20 | 16,735,001 | 144 | 27,934,389 | 1216 | 25,676,056 | 828 | 6,252,050 |
| 2003 | 2341 | 77323962 | 17 | 16,768,712 | 135 | 27,932,493 | 1346 | 26,323,318 | 843 | 6,299,439 |
| 2004 | 2434 | 78044525 | 13 | 16,763,691 | 139 | 28,008,673 | 1409 | 26,947,825 | 873 | 6,324,336 |
| 2005 | 2478 | 78545195 | 14 | 16,655,645 | 146 | 28,038,343 | 1413 | 27,469,344 | 905 | 6,381,863 |
| 2006 | 2339 | 79131483 | 22 | 16,576,106 | 96 | 28,171,430 | 1422 | 27,951,499 | 799 | 6,432,448 |
| 2007 | 2595 | 79874119 | 31 | 16,595,177 | 122 | 28,343,811 | 1529 | 28,435,048 | 913 | 6,500,083 |
| 2008 | 2556 | 80716323 | 26 | 16,660,990 | 114 | 28,532,744 | 1513 | 28,932,257 | 903 | 6,590,332 |
| 2009 | 2636 | 81524852 | 21 | 16,705,611 | 111 | 28,681,979 | 1539 | 29,445,174 | 965 | 6,692,088 |
| 2010 | 2674 | 82300337 | 19 | 16,746,484 | 141 | 28,748,383 | 1620 | 30,001,339 | 894 | 6,804,131 |
| 2011 | 2724 | 83029250 | 26 | 16,755,498 | 148 | 28,869,445 | 1658 | 30,480,398 | 892 | 6,923,909 |
| 2012 | 2923 | 83710141 | 26 | 16,737,466 | 153 | 29,044,700 | 1740 | 30,848,024 | 1004 | 7,079,951 |
| 2013 | 2944 | 84358084 | 34 | 16,728,927 | 131 | 29,245,348 | 1753 | 31,098,977 | 1026 | 7,284,832 |
| 2014 | 2911 | 85031165 | 27 | 16,722,537 | 137 | 29,486,098 | 1761 | 31,341,911 | 986 | 7,480,619 |
| 2015 | 3037 | 85709493 | 22 | 16,699,583 | 131 | 29,730,941 | 1792 | 31,606,184 | 1092 | 7,672,785 |
| 2016 | 3073 | 86351333 | 20 | 16,686,706 | 150 | 29,985,813 | 1805 | 31,817,889 | 1098 | 7,860,925 |
| 2017 | 3148 | 86894658 | 28 | 16,673,694 | 181 | 30,158,515 | 1839 | 31,850,736 | 1100 | 8,211,713 |
| 2018 | 3256 | 87296824 | 30 | 16,607,869 | 154 | 30,278,197 | 1913 | 31,898,031 | 1159 | 8,512,727 |
| 2019 | 3313 | 87587336 | 18 | 16,495,886 | 167 | 30,352,056 | 1899 | 31,935,802 | 1229 | 8,803,592 |
| 2020 | 3459 | 87746852 | 17 | 16,344,103 | 184 | 30,318,059 | 1928 | 32,007,523 | 1330 | 9,077,167 |
| 2021 | 3294 | 87497595 | 16 | 16,084,236 | 127 | 30,092,652 | 1826 | 31,991,254 | 1325 | 9,329,453 |
