# Supplementary material for: Evaluating short-term survivors of glioblastoma: A proposal based on SEER registry data
Source: Neurooncol Adv. 2025 Feb 9;7(1):vdaf036. doi: 10.1093/noajnl/vdaf036 (PMC12080546; doi:10.1093/noajnl/vdaf036)
Supplement: vdaf036_suppl_Supplementary_Table_S2 [file vdaf036_suppl_supplementary_table_s2.docx]

**Supplemental table 2. Trends in the number of decedents from glioblastoma and estimated number of population by age groups**

|  | **All** | | **0-14 years of age** | | **15-39 years of age** | | **40-69 years of age** | | **70+ years of age** | |
| --- | --- | --- | --- | --- | --- | --- | --- | --- | --- | --- |
| **Year** | **Decedents** | **Population** | **Patients** | **Population** | **Patients** | **Population** | **Patients** | **Population** | **Patients** | **Population** |
| 2000 | 895 | 74952547 | 5 | 16638605 | 24 | 27808930 | 397 | 24359021 | 469 | 6145991 |
| 2001 | 1646 | 75823654 | 5 | 16698155 | 64 | 27896701 | 863 | 25024570 | 714 | 6204228 |
| 2002 | 1909 | 76597496 | 16 | 16735001 | 61 | 27934389 | 1036 | 25676056 | 796 | 6252050 |
| 2003 | 2023 | 77323962 | 14 | 16768712 | 81 | 27932493 | 1149 | 26323318 | 779 | 6299439 |
| 2004 | 1962 | 78044525 | 11 | 16763691 | 80 | 28008673 | 1068 | 26947825 | 803 | 6324336 |
| 2005 | 2171 | 78545195 | 6 | 16655645 | 93 | 28038343 | 1212 | 27469344 | 860 | 6381863 |
| 2006 | 2027 | 79131483 | 11 | 16576106 | 86 | 28171430 | 1157 | 27951499 | 773 | 6432448 |
| 2007 | 2126 | 79874119 | 18 | 16595177 | 68 | 28343811 | 1206 | 28435048 | 834 | 6500083 |
| 2008 | 2171 | 80716323 | 19 | 16660990 | 73 | 28532744 | 1241 | 28932257 | 838 | 6590332 |
| 2009 | 2271 | 81524852 | 15 | 16705611 | 72 | 28681979 | 1289 | 29445174 | 895 | 6692088 |
| 2010 | 2290 | 82300337 | 11 | 16746484 | 74 | 28748383 | 1343 | 30001339 | 862 | 6804131 |
| 2011 | 2363 | 83029250 | 22 | 16755498 | 92 | 28869445 | 1375 | 30480398 | 874 | 6923909 |
| 2012 | 2468 | 83710141 | 16 | 16737466 | 91 | 29044700 | 1452 | 30848024 | 909 | 7079951 |
| 2013 | 2475 | 84358084 | 21 | 16728927 | 97 | 29245348 | 1437 | 31098977 | 920 | 7284832 |
| 2014 | 2629 | 85031165 | 19 | 16722537 | 95 | 29486098 | 1509 | 31341911 | 1006 | 7480619 |
| 2015 | 2661 | 85709493 | 23 | 16699583 | 98 | 29730941 | 1526 | 31606184 | 1014 | 7672785 |
| 2016 | 2828 | 86351333 | 18 | 16686706 | 86 | 29985813 | 1621 | 31817889 | 1103 | 7860925 |
| 2017 | 2688 | 86894658 | 17 | 16673694 | 82 | 30158515 | 1528 | 31850736 | 1061 | 8211713 |
| 2018 | 2835 | 87296824 | 19 | 16607869 | 113 | 30278197 | 1567 | 31898031 | 1136 | 8512727 |
| 2019 | 2833 | 87587336 | 11 | 16495886 | 111 | 30352056 | 1594 | 31935802 | 1117 | 8803592 |
| 2020 | 3025 | 87746852 | 6 | 16344103 | 75 | 30318059 | 1661 | 32007523 | 1283 | 9077167 |
| 2021 | 2878 | 87497595 | 13 | 16084236 | 85 | 30092652 | 1533 | 31991254 | 1247 | 9329453 |
