# Supplementary material for: Evaluating short-term survivors of glioblastoma: A proposal based on SEER registry data
Source: Neurooncol Adv. 2025 Feb 9;7(1):vdaf036. doi: 10.1093/noajnl/vdaf036 (PMC12080546; doi:10.1093/noajnl/vdaf036)
Supplement: vdaf036_suppl_Supplementary_Table_S3 [file vdaf036_suppl_supplementary_table_s3.docx]

**Supplemental table 3. Trends in the number of patients and decedents from glioblastoma and estimated number of population by sex**

|  | **Female** | | | | **Male** | | | |
| --- | --- | --- | --- | --- | --- | --- | --- | --- |
|  | **Incidence** | | **Mortality** | | **Incidence** | | **Mortality** | |
| **Year** | **Patients** | **Population** | **Decedents** | **Population** | **Patients** | **Population** | **Decedents** | **Population** |
| 2000 | 874 | 37937902 | 380 | 37937902 | 1255 | 37,014,645 | 515 | 37014645 |
| 2001 | 931 | 38361776 | 703 | 38361776 | 1212 | 37,461,878 | 943 | 37461878 |
| 2002 | 902 | 38756152 | 795 | 38756152 | 1306 | 37,841,344 | 1114 | 37841344 |
| 2003 | 989 | 39152130 | 880 | 39152130 | 1352 | 38,171,832 | 1143 | 38171832 |
| 2004 | 1032 | 39501584 | 846 | 39501584 | 1402 | 38,542,941 | 1116 | 38542941 |
| 2005 | 1046 | 39749598 | 883 | 39749598 | 1432 | 38,795,597 | 1288 | 38795597 |
| 2006 | 987 | 40040108 | 848 | 40040108 | 1352 | 39,091,375 | 1179 | 39091375 |
| 2007 | 1098 | 40417426 | 902 | 40417426 | 1497 | 39,456,693 | 1224 | 39456693 |
| 2008 | 1059 | 40842092 | 902 | 40842092 | 1497 | 39,874,231 | 1269 | 39874231 |
| 2009 | 1112 | 41249156 | 962 | 41249156 | 1524 | 40,275,696 | 1309 | 40275696 |
| 2010 | 1159 | 41644278 | 982 | 41644278 | 1515 | 40,656,059 | 1308 | 40656059 |
| 2011 | 1121 | 41981998 | 993 | 41981998 | 1603 | 41,047,252 | 1370 | 41047252 |
| 2012 | 1226 | 42285669 | 998 | 42285669 | 1697 | 41,424,472 | 1470 | 41424472 |
| 2013 | 1248 | 42578045 | 1065 | 42578045 | 1696 | 41,780,039 | 1410 | 41780039 |
| 2014 | 1192 | 42877998 | 1101 | 42877998 | 1719 | 42,153,167 | 1528 | 42153167 |
| 2015 | 1307 | 43179021 | 1104 | 43179021 | 1730 | 42,530,472 | 1557 | 42530472 |
| 2016 | 1299 | 43464439 | 1165 | 43464439 | 1774 | 42,886,894 | 1663 | 42886894 |
| 2017 | 1275 | 43698643 | 1122 | 43698643 | 1873 | 43,196,015 | 1566 | 43196015 |
| 2018 | 1338 | 43864726 | 1156 | 43864726 | 1918 | 43,432,098 | 1679 | 43432098 |
| 2019 | 1369 | 43976470 | 1162 | 43976470 | 1944 | 43,610,866 | 1671 | 43610866 |
| 2020 | 1412 | 44034978 | 1223 | 44034978 | 2047 | 43,711,874 | 1802 | 43711874 |
| 2021 | 1422 | 43935904 | 1217 | 43935904 | 1872 | 43,561,691 | 1661 | 43561691 |
