# Supplementary material for: Evaluating short-term survivors of glioblastoma: A proposal based on SEER registry data
Source: Neurooncol Adv. 2025 Feb 9;7(1):vdaf036. doi: 10.1093/noajnl/vdaf036 (PMC12080546; doi:10.1093/noajnl/vdaf036)
Supplement: vdaf036_suppl_Supplementary_Table_S4 [file vdaf036_suppl_supplementary_table_s4.docx]

**Supplemental table 4. Trends in the number of patients and decedents from glioblastoma and estimated number of population by race/ethnicity**

|  | **Non-Hispanic White** | | | | **Non-Hispanic Black** | | | | **Non-Hispanic Asian and Pacific Islander** | | | | **Hispanic** | | | |
| --- | --- | --- | --- | --- | --- | --- | --- | --- | --- | --- | --- | --- | --- | --- | --- | --- |
|  | **Incidence** | | **Mortality** | | **Incidence** | | **Mortality** | | **Incidence** | | **Mortality** | | **Incidence** | | **Mortality** | |
| **Year** | **Patients** | **Population** | **Decedents** | **Population** | **Patients** | **Population** | **Decedents** | **Population** | **Patients** | **Population** | **Decedents** | **Population** | **Patients** | **Population** | **Decedents** | **Population** |
| 2000 | 1788 | 45326926 | 766 | 45326926 | 112 | 8273982 | 52 | 8273982 | 63 | 6194772 | 20 | 6194772 | 159 | 14479774 | 56 | 14479774 |
| 2001 | 1760 | 45367690 | 1373 | 45367690 | 109 | 8371873 | 90 | 8371873 | 75 | 6423693 | 47 | 6423693 | 189 | 14979207 | 129 | 14979207 |
| 2002 | 1806 | 45342332 | 1594 | 45342332 | 94 | 8463909 | 81 | 8463909 | 93 | 6636287 | 77 | 6636287 | 203 | 15470807 | 147 | 15470807 |
| 2003 | 1912 | 45297717 | 1696 | 45297717 | 122 | 8543851 | 81 | 8543851 | 90 | 6837470 | 71 | 6837470 | 210 | 15958379 | 170 | 15958379 |
| 2004 | 2018 | 45247272 | 1625 | 45247272 | 109 | 8639532 | 103 | 8639532 | 85 | 7034430 | 56 | 7034430 | 216 | 16433631 | 168 | 16433631 |
| 2005 | 2019 | 45087676 | 1808 | 45087676 | 112 | 8645709 | 103 | 8645709 | 99 | 7232214 | 77 | 7232214 | 237 | 16888372 | 179 | 16888372 |
| 2006 | 1888 | 44983875 | 1673 | 44983875 | 120 | 8695868 | 91 | 8695868 | 90 | 7427370 | 81 | 7427370 | 235 | 17332449 | 178 | 17332449 |
| 2007 | 2090 | 44934952 | 1787 | 44934952 | 131 | 8834094 | 88 | 8834094 | 117 | 7615000 | 72 | 7615000 | 246 | 17796107 | 172 | 17796107 |
| 2008 | 2047 | 44935583 | 1765 | 44935583 | 124 | 8958258 | 109 | 8958258 | 103 | 7817298 | 80 | 7817298 | 268 | 18307958 | 205 | 18307958 |
| 2009 | 2148 | 44927032 | 1882 | 44927032 | 152 | 9073248 | 100 | 9073248 | 94 | 8004127 | 73 | 8004127 | 227 | 18819129 | 210 | 18819129 |
| 2010 | 2087 | 44934208 | 1851 | 44934208 | 146 | 9176912 | 128 | 9176912 | 132 | 8190964 | 93 | 8190964 | 295 | 19293975 | 206 | 19293975 |
| 2011 | 2141 | 44982918 | 1894 | 44982918 | 155 | 9287599 | 121 | 9287599 | 132 | 8405235 | 105 | 8405235 | 278 | 19644676 | 227 | 19644676 |
| 2012 | 2306 | 45030785 | 1980 | 45030785 | 159 | 9389378 | 129 | 9389378 | 150 | 8620131 | 114 | 8620131 | 293 | 19956497 | 236 | 19956497 |
| 2013 | 2278 | 45041216 | 1974 | 45041216 | 168 | 9484670 | 114 | 9484670 | 156 | 8847237 | 122 | 8847237 | 327 | 20267384 | 248 | 20267384 |
| 2014 | 2258 | 45057062 | 2062 | 45057062 | 155 | 9589842 | 151 | 9589842 | 150 | 9084812 | 129 | 9084812 | 337 | 20577930 | 275 | 20577930 |
| 2015 | 2339 | 45053444 | 2081 | 45053444 | 161 | 9694346 | 145 | 9694346 | 149 | 9346259 | 138 | 9346259 | 374 | 20890195 | 287 | 20890195 |
| 2016 | 2340 | 45023144 | 2234 | 45023144 | 161 | 9800119 | 148 | 9800119 | 193 | 9591442 | 125 | 9591442 | 359 | 21208899 | 305 | 21208899 |
| 2017 | 2369 | 44929749 | 2093 | 44929749 | 193 | 9904163 | 134 | 9904163 | 150 | 9824082 | 135 | 9824082 | 410 | 21506386 | 312 | 21506386 |
| 2018 | 2436 | 44787551 | 2190 | 44787551 | 190 | 9996275 | 161 | 9996275 | 180 | 10007743 | 149 | 10007743 | 431 | 21775322 | 316 | 21775322 |
| 2019 | 2451 | 44596034 | 2185 | 44596034 | 190 | 10091023 | 175 | 10091023 | 187 | 10164135 | 134 | 10164135 | 456 | 22005796 | 324 | 22005796 |
| 2020 | 2535 | 44341456 | 2265 | 44341456 | 211 | 10160988 | 156 | 10160988 | 226 | 10285199 | 179 | 10285199 | 458 | 22227158 | 398 | 22227158 |
| 2021 | 2427 | 43869312 | 2155 | 43869312 | 186 | 10179768 | 159 | 10179768 | 180 | 10346003 | 154 | 10346003 | 464 | 22371415 | 377 | 22371415 |
