# Supplementary material for: Evaluating short-term survivors of glioblastoma: A proposal based on SEER registry data
Source: Neurooncol Adv. 2025 Feb 9;7(1):vdaf036. doi: 10.1093/noajnl/vdaf036 (PMC12080546; doi:10.1093/noajnl/vdaf036)
Supplement: vdaf036_suppl_Supplementary_Table_S5 [file vdaf036_suppl_supplementary_table_s5.docx]

**Supplemental table 5. Distribution of the number of patients and decedents from glioblastomas and estimated number of population**

|  | **All** | | | | **Short-term survivors** | | | | **Long-term survivors** | | | |
| --- | --- | --- | --- | --- | --- | --- | --- | --- | --- | --- | --- | --- |
|  | **Incidence** | | **Mortality** | | **Incidence** | | **Mortality** | | **Incidence** | | **Mortality** | |
| **Age groups (years)** | **Patients** | **Population** | **Decedents** | **Population** | **Patients** | **Population** | **Decedents** | **Population** | **Patients** | **Population** | **Decedents** | **Population** |
| 00 years | 47 | 23696350 | 19 | 23696350 | 23 | 23696350 | 19 | 23696350 | 8 | 23696350 | 0 | 23696350 |
| 01-04 years | 71 | 95290005 | 32 | 95290005 | 19 | 95290005 | 16 | 95290005 | 11 | 95290005 | 0 | 95290005 |
| 05-09 years | 154 | 120770298 | 114 | 120770298 | 38 | 120770298 | 39 | 120770298 | 9 | 120770298 | 2 | 120770298 |
| 10-14 years | 206 | 126324029 | 151 | 126324029 | 38 | 126324029 | 38 | 126324029 | 30 | 126324029 | 7 | 126324029 |
| 15-19 years | 259 | 127486167 | 188 | 127486167 | 45 | 127486167 | 41 | 127486167 | 34 | 127486167 | 17 | 127486167 |
| 20-24 years | 338 | 128548015 | 209 | 128548015 | 42 | 128548015 | 42 | 128548015 | 78 | 128548015 | 23 | 128548015 |
| 25-29 years | 544 | 127725903 | 281 | 127725903 | 67 | 127725903 | 58 | 127725903 | 139 | 127725903 | 45 | 127725903 |
| 30-34 years | 757 | 126895737 | 416 | 126895737 | 111 | 126895737 | 102 | 126895737 | 176 | 126895737 | 74 | 126895737 |
| 35-39 years | 1160 | 127003878 | 707 | 127003878 | 180 | 127003878 | 170 | 127003878 | 233 | 127003878 | 99 | 127003878 |
| 40-44 years | 1986 | 127048383 | 1332 | 127048383 | 382 | 127048383 | 343 | 127048383 | 282 | 127048383 | 136 | 127048383 |
| 45-49 years | 3410 | 125993875 | 2459 | 125993875 | 739 | 125993875 | 663 | 125993875 | 385 | 125993875 | 225 | 125993875 |
| 50-54 years | 5430 | 121002686 | 4074 | 121002686 | 1418 | 121002686 | 1251 | 121002686 | 546 | 121002686 | 314 | 121002686 |
| 55-59 years | 7507 | 109505547 | 6084 | 109505547 | 2226 | 109505547 | 2030 | 109505547 | 629 | 109505547 | 421 | 109505547 |
| 60-64 years | 8513 | 91350141 | 7312 | 91350141 | 3094 | 91350141 | 2833 | 91350141 | 552 | 91350141 | 463 | 91350141 |
| 65-69 years | 8491 | 72541548 | 7503 | 72541548 | 3814 | 72541548 | 3458 | 72541548 | 410 | 72541548 | 407 | 72541548 |
| 70-74 years | 8071 | 56189183 | 7339 | 56189183 | 4335 | 56189183 | 4007 | 56189183 | 230 | 56189183 | 287 | 56189183 |
| 75-79 years | 6592 | 42306775 | 6111 | 42306775 | 4266 | 42306775 | 3923 | 42306775 | 118 | 42306775 | 165 | 42306775 |
| 80-84 years | 4373 | 30582863 | 4201 | 30582863 | 3364 | 30582863 | 3148 | 30582863 | 40 | 30582863 | 70 | 30582863 |
| 85+ years | 2706 | 29785841 | 2642 | 29785841 | 2309 | 29785841 | 2190 | 29785841 | 11 | 29785841 | 26 | 29785841 |
