# Supplementary material for: Evaluating short-term survivors of glioblastoma: A proposal based on SEER registry data
Source: Neurooncol Adv. 2025 Feb 9;7(1):vdaf036. doi: 10.1093/noajnl/vdaf036 (PMC12080546; doi:10.1093/noajnl/vdaf036)
Supplement: vdaf036_suppl_Supplementary_Table_S6 [file vdaf036_suppl_supplementary_table_s6.docx]

**Supplemental Table 6. Demographic features of survival data within short-time survivors and long-term survivors.**

|  |  | **Short-term survivor** | **Long-term survivor** |  |  |
| --- | --- | --- | --- | --- | --- |
| **Total cases** | | **16625** | **4197** | **p-value** | **OR (95%CI)** |
| Dead case | | 16625 (100%) | 2919 (69.5%) | - | - |
| Cause of death | Glioblastoma | 15434 (92.8%) | 2675 (91.6%) | 0.026 | 0.84 (0.73-0.98) |
| (/all dead cases) | All other CODs | 1191 (7.2%) | 244 (8.4%) |  |  |
|  | Diseases of heart | 290 (1.9%) | 37 (1.4%) | 0.072 | 1.38 (0.99-1.94) |
|  | Pneumonia and Influenza | 40 (0.3%) | 7 (0.3%) | 1.000 | 1.00 (0.46-2.29) |
|  | Cerebrovascular diseases | 93 (0.6%) | 24 (0.9%) | 0.091 | 0.68 (0.44-1.08) |
|  | Accidents and adverse events | 30 (0.2%) | 13 (0.5%) | 0.009 | 0.40 (0.21-0.78) |
|  | Other Infectious and Parasitic Diseases including HIV | 27 (0.2%) | 1 (0.0%) | 0.111 | 4.75 (0.83-48.99) |
|  | Others | 711 (4.6%) | 162 (6.1%) | 0.003 | 0.76 (0.64-0.91) |
| CODs, causes of death. | | | | | |
