# Supplementary material for: Evaluating short-term survivors of glioblastoma: A proposal based on SEER registry data
Source: Neurooncol Adv. 2025 Feb 9;7(1):vdaf036. doi: 10.1093/noajnl/vdaf036 (PMC12080546; doi:10.1093/noajnl/vdaf036)
Supplement: vdaf036_suppl_Supplementary_Table_S7 [file vdaf036_suppl_supplementary_table_s7.docx]

**Supplemental Table 7. Trends in age-adjusted incidence in glioblastoma by age groups**

|  | **All** | | **0-14 years of age** | | **15-39 years of age** | | **40-69 years of age** | | **70+ years of age** | |
| --- | --- | --- | --- | --- | --- | --- | --- | --- | --- | --- |
| **Year** | **AAIR (95% CI))** | **AAPC** | **AAIR (95% CI))** | **AAPC** | **AAIR (95% CI))** | **AAPC** | **AAIR (95% CI))** | **AAPC** | **AAIR (95% CI))** | **AAPC** |
| 2000 | 3.06 (2.93, 3.18) | 0.20 (0.01, 0.42) | 0.09 (0.05, 0.15) | 1.44 (-1.16, 4.41) | 0.46 (0.38, 0.54) | 0.85 (-0.04, 1.87) | 4.98 (4.70, 5.27) | -0.01 (-.021, 0.20) | 12.93 (12.05, 13.87) | 0.38 (0.02, 0.79) |
| 2001 | 3.02 (2.90, 3.15) |  | 0.10 (0.05, 0.15) |  | 0.44 (0.37, 0.53) |  | 4.98 (4.70, 5.27) |  | 12.61 (11.74, 13.53) |  |
| 2002 | 3.06 (2.93, 3.19) |  | 0.12 (0.07, 0.18) |  | 0.52 (0.44, 0.62) |  | 4.80 (4.53, 5.08) |  | 13.30 (12.41, 14.23) |  |
| 2003 | 3.17 (3.05, 3.31) |  | 0.10 (0.06, 0.16) |  | 0.49 (0.41, 0.58) |  | 5.14 (4.87, 5.43) |  | 13.46 (12.57, 14.40) |  |
| 2004 | 3.24 (3.11, 3.37) |  | 0.08 (0.04, 0.13) |  | 0.51 (0.43, 0.60) |  | 5.22 (4.95, 5.50) |  | 13.81 (12.91, 14.76) |  |
| 2005 | 3.25 (3.12, 3.38) |  | 0.08 (0.05, 0.14) |  | 0.54 (0.45, 0.63) |  | 5.09 (4.83, 5.36) |  | 14.31 (13.39, 15.27) |  |
| 2006 | 2.99 (2.86, 3.11) |  | 0.13 (0.08, 0.20) |  | 0.35 (0.29, 0.43) |  | 4.97 (4.71, 5.23) |  | 12.52 (11.66, 13.42) |  |
| 2007 | 3.26 (3.14, 3.39) |  | 0.19 (0.13, 0.27) |  | 0.45 (0.37, 0.53) |  | 5.19 (4.93, 5.46) |  | 14.21 (13.30, 15.16) |  |
| 2008 | 3.15 (3.03, 3.28) |  | 0.16 (0.10, 0.23) |  | 0.42 (0.35, 0.50) |  | 4.99 (4.74, 5.25) |  | 13.90 (13.00, 14.84) |  |
| 2009 | 3.16 (3.04, 3.29) |  | 0.13 (0.08, 0.19) |  | 0.41 (0.33, 0.49) |  | 4.89 (4.64, 5.14) |  | 14.54 (13.63, 15.49) |  |
| 2010 | 3.13 (3.01, 3.25) |  | 0.11 (0.07, 0.18) |  | 0.51 (0.43, 0.60) |  | 5.02 (4.78, 5.27) |  | 13.34 (12.48, 14.25) |  |
| 2011 | 3.13 (3.01, 3.25) |  | 0.16 (0.10, 0.23) |  | 0.54 (0.45, 0.63) |  | 5.01 (4.77, 5.26) |  | 13.12 (12.27, 14.01) |  |
| 2012 | 3.28 (3.16, 3.40) |  | 0.16 (0.10, 0.23) |  | 0.54 (0.46, 0.64) |  | 5.08 (4.84, 5.32) |  | 14.47 (13.58, 15.39) |  |
| 2013 | 3.25 (3.13, 3.37) |  | 0.20 (0.14, 0.28) |  | 0.48 (0.40, 0.57) |  | 5.06 (4.83, 5.31) |  | 14.33 (13.46, 15.24) |  |
| 2014 | 3.11 (3.00, 3.23) |  | 0.16 (0.11, 0.24) |  | 0.48 (0.40, 0.57) |  | 4.97 (4.74, 5.21) |  | 13.31 (12.49, 14.17) |  |
| 2015 | 3.22 (3.10, 3.33) |  | 0.13 (0.08, 0.20) |  | 0.47 (0.39, 0.56) |  | 4.99 (4.75, 5.23) |  | 14.48 (13.63, 15.37) |  |
| 2016 | 3.18 (3.07, 3.30) |  | 0.12 (0.07, 0.18) |  | 0.52 (0.44, 0.61) |  | 4.90 (4.67, 5.13) |  | 14.26 (13.42, 15.13) |  |
| 2017 | 3.17 (3.06, 3.29) |  | 0.17 (0.11, 0.24) |  | 0.62 (0.53, 0.71) |  | 4.94 (4.71, 5.18) |  | 13.52 (12.73, 14.35) |  |
| 2018 | 3.23 (3.12, 3.35) |  | 0.18 (0.12, 0.26) |  | 0.53 (0.45, 0.62) |  | 5.15 (4.92, 5.39) |  | 13.75 (12.97, 14.57) |  |
| 2019 | 3.24 (3.13, 3.36) |  | 0.11 (0.06, 0.17) |  | 0.56 (0.48, 0.65) |  | 5.10 (4.87, 5.34) |  | 14.08 (13.30, 14.89) |  |
| 2020 | 3.33 (3.22, 3.44) |  | 0.10 (0.06, 0.16) |  | 0.62 (0.53, 0.71) |  | 5.12 (4.89, 5.36) |  | 14.71 (13.93, 15.53) |  |
| 2021 | 3.13 (3.03, 3.24) |  | 0.10 (0.06, 0.16) |  | 0.43 (0.36, 0.51) |  | 4.86 (4.63, 5.10) |  | 14.29 (13.53, 15.09) |  |
| AAIR, age-adjusted incidence rate; AAPC, average annual percent change; CI, confidence interval. | | | | | |  |  |  |  |  |
