# Supplementary material for: Evaluating short-term survivors of glioblastoma: A proposal based on SEER registry data
Source: Neurooncol Adv. 2025 Feb 9;7(1):vdaf036. doi: 10.1093/noajnl/vdaf036 (PMC12080546; doi:10.1093/noajnl/vdaf036)
Supplement: vdaf036_suppl_Supplementary_Table_S8 [file vdaf036_suppl_supplementary_table_s8.docx]

**Supplemental Table 8. Trends in age-adjusted mortality in glioblastoma by age groups**

|  | **All** | | **0-14 years of age** | | **15-39 years of age** | | **40-69 years of age** | | **70+ years of age** | |
| --- | --- | --- | --- | --- | --- | --- | --- | --- | --- | --- |
| **Year** | **AAMR (95% CI)** | **AAPC** | **AAMR (95% CI)** | **AAPC** | **AAMR (95% CI)** | **AAPC** | **AAMR (95% CI)** | **AAPC** | **AAMR (95% CI)** | **AAPC** |
| 2000 | 1.30 (1.22, 1.39) | 0.76 (-0.23, 1.87) | 0.03 (0.01, 0.07) | 2.02 (-1.28, 6.14) | 0.09 (0.06, 0.13) | 1.58 (0.08, 3.32) | 1.67 (1.51, 1.84) | 0.60  (-0.55, 1.89) | 7.62 (6.95, 8.34) | 0.85  (0.10, 1.69) |
| 2001 | 2.34 (2.23, 2.46) |  | 0.03 (0.01, 0.07) |  | 0.23 (0.18, 0.30) |  | 3.53 (3.30, 3.77) |  | 11.50 (10.67, 12.37) |  |
| 2002 | 2.65 (2.54, 2.78) |  | 0.09 (0.05, 0.15) |  | 0.22 (0.17, 0.28) |  | 4.08 (3.84, 4.34) |  | 12.76 (11.89, 13.68) |  |
| 2003 | 2.76 (2.64, 2.89) |  | 0.08 (0.05, 0.14) |  | 0.30 (0.24, 0.37) |  | 4.41 (4.16, 4.67) |  | 12.45 (11.59, 13.36) |  |
| 2004 | 2.63 (2.51, 2.75) |  | 0.07 (0.03, 0.12) |  | 0.30 (0.23, 0.37) |  | 3.95 (3.72, 4.20) |  | 12.72 (11.85, 13.63) |  |
| 2005 | 2.85 (2.74, 2.98) |  | 0.03 (0.01, 0.08) |  | 0.34 (0.28, 0.42) |  | 4.37 (4.12, 4.62) |  | 13.56 (12.67, 14.50) |  |
| 2006 | 2.60 (2.49, 2.72) |  | 0.07 (0.03, 0.12) |  | 0.31 (0.25, 0.39) |  | 4.04 (3.81, 4.28) |  | 12.08 (11.24, 12.97) |  |
| 2007 | 2.69 (2.58, 2.81) |  | 0.11 (0.07, 0.17) |  | 0.25 (0.19, 0.32) |  | 4.10 (3.87, 4.34) |  | 12.95 (12.08, 13.86) |  |
| 2008 | 2.68 (2.56, 2.79) |  | 0.12 (0.07, 0.18) |  | 0.27 (0.21, 0.33) |  | 4.06 (3.84, 4.29) |  | 12.86 (12.01, 13.77) |  |
| 2009 | 2.75 (2.63, 2.86) |  | 0.09 (0.05, 0.15) |  | 0.26 (0.21, 0.33) |  | 4.11 (3.89, 4.34) |  | 13.49 (12.62, 14.41) |  |
| 2010 | 2.69 (2.58, 2.80) |  | 0.07 (0.03, 0.12) |  | 0.27 (0.21, 0.34) |  | 4.12 (3.91, 4.34) |  | 12.83 (11.99, 13.72) |  |
| 2011 | 2.72 (2.61, 2.83) |  | 0.13 (0.08, 0.20) |  | 0.33 (0.27, 0.41) |  | 4.11 (3.89, 4.33) |  | 12.81 (11.97, 13.69) |  |
| 2012 | 2.77 (2.66, 2.88) |  | 0.10 (0.06, 0.16) |  | 0.33 (0.26, 0.40) |  | 4.22 (4.01, 4.45) |  | 13.05 (12.21, 13.93) |  |
| 2013 | 2.72 (2.62, 2.84) |  | 0.13 (0.08, 0.19) |  | 0.35 (0.28, 0.43) |  | 4.11 (3.90, 4.33) |  | 12.84 (12.02, 13.70) |  |
| 2014 | 2.82 (2.71, 2.93) |  | 0.11 (0.07, 0.18) |  | 0.33 (0.27, 0.41) |  | 4.21 (4.00, 4.43) |  | 13.61 (12.77, 14.48) |  |
| 2015 | 2.79 (2.69, 2.90) |  | 0.14 (0.09, 0.21) |  | 0.35 (0.28, 0.43) |  | 4.17 (3.96, 4.39) |  | 13.32 (12.51, 14.17) |  |
| 2016 | 2.92 (2.82, 3.04) |  | 0.11 (0.06, 0.17) |  | 0.30 (0.24, 0.38) |  | 4.36 (4.15, 4.58) |  | 14.31 (13.47, 15.18) |  |
| 2017 | 2.72 (2.61, 2.82) |  | 0.10 (0.06, 0.16) |  | 0.28 (0.22, 0.35) |  | 4.09 (3.88, 4.30) |  | 13.16 (12.37, 13.98) |  |
| 2018 | 2.80 (2.69, 2.91) |  | 0.11 (0.07, 0.18) |  | 0.39 (0.32, 0.47) |  | 4.13 (3.92, 4.34) |  | 13.47 (12.69, 14.28) |  |
| 2019 | 2.74 (2.64, 2.84) |  | 0.07 (0.03, 0.12) |  | 0.38 (0.31, 0.46) |  | 4.16 (3.95, 4.37) |  | 12.85 (12.11, 13.64) |  |
| 2020 | 2.88 (2.78, 2.99) |  | 0.04 (0.01, 0.08) |  | 0.25 (0.20, 0.32) |  | 4.36 (4.15, 4.58) |  | 14.20 (13.43, 15.00) |  |
| 2021 | 2.71 (2.61, 2.82) |  | 0.08 (0.04, 0.14) |  | 0.29 (0.23, 0.35) |  | 4.00 (3.80, 4.22) |  | 13.47 (12.72, 14.24) |  |
| AAMR, age-adjusted mortality rate; AAPC, average annual percent change; CI, confidence interval. | | | | | | | | | | |
