# Supplementary material for: Evaluating short-term survivors of glioblastoma: A proposal based on SEER registry data
Source: Neurooncol Adv. 2025 Feb 9;7(1):vdaf036. doi: 10.1093/noajnl/vdaf036 (PMC12080546; doi:10.1093/noajnl/vdaf036)
Supplement: vdaf036_suppl_Supplementary_Table_S9 [file vdaf036_suppl_supplementary_table_s9.docx]

**Supplemental Table 9. Trends in age-adjusted incidence and mortality in glioblastoma by sex**

|  | **Female** | | | | **Male** | | | |
| --- | --- | --- | --- | --- | --- | --- | --- | --- |
|  | **Incidence** | | **Mortality** | | **Incidence** | | **Mortality** | |
| **Year** | **AAIR (95% CI))** | **AAPC** | **AAMR (95% CI)** | **AAPC** | **AAIR (95% CI))** | **AAPC** | **AAMR (95% CI)** | **AAPC** |
| 2000 | 2.30 (2.15, 2.46) | 0.30 (-0.01, 0.65) | 0.99 (0.89, 1.09) | 0.77  (-0.23, 1.95) | 4.01 (3.79, 4.25) | 0.05 (-0.14, 0.27) | 1.73 (1.58, 1.89) | 0.64 (-0.26, 1.70) |
| 2001 | 2.41 (2.25, 2.57) |  | 1.80 (1.67, 1.94) |  | 3.76 (3.55, 3.98) |  | 3.00 (2.81, 3.19) |  |
| 2002 | 2.30 (2.15, 2.45) |  | 2.02 (1.88, 2.16) |  | 4.01 (3.79, 4.24) |  | 3.46 (3.26, 3.67) |  |
| 2003 | 2.47 (2.32, 2.63) |  | 2.20 (2.06, 2.35) |  | 4.02 (3.81, 4.25) |  | 3.46 (3.26, 3.67) |  |
| 2004 | 2.52 (2.37, 2.68) |  | 2.07 (1.93, 2.21) |  | 4.05 (3.84, 4.28) |  | 3.30 (3.10, 3.50) |  |
| 2005 | 2.52 (2.37, 2.68) |  | 2.12 (1.98, 2.26) |  | 4.12 (3.90, 4.34) |  | 3.74 (3.53, 3.95) |  |
| 2006 | 2.34 (2.20, 2.50) |  | 2.01 (1.87, 2.15) |  | 3.76 (3.56, 3.97) |  | 3.34 (3.15, 3.54) |  |
| 2007 | 2.55 (2.40, 2.71) |  | 2.10 (1.97, 2.25) |  | 4.09 (3.89, 4.31) |  | 3.38 (3.19, 3.58) |  |
| 2008 | 2.42 (2.27, 2.57) |  | 2.05 (1.92, 2.19) |  | 4.01 (3.81, 4.22) |  | 3.42 (3.23, 3.62) |  |
| 2009 | 2.48 (2.33, 2.63) |  | 2.15 (2.01, 2.29) |  | 3.97 (3.77, 4.18) |  | 3.47 (3.28, 3.67) |  |
| 2010 | 2.52 (2.37, 2.67) |  | 2.14 (2.00, 2.28) |  | 3.85 (3.66, 4.06) |  | 3.34 (3.16, 3.53) |  |
| 2011 | 2.41 (2.27, 2.56) |  | 2.13 (1.99, 2.27) |  | 3.98 (3.78, 4.18) |  | 3.43 (3.24, 3.62) |  |
| 2012 | 2.58 (2.43, 2.73) |  | 2.08 (1.95, 2.21) |  | 4.12 (3.92, 4.32) |  | 3.60 (3.41, 3.79) |  |
| 2013 | 2.56 (2.42, 2.71) |  | 2.18 (2.05, 2.32) |  | 4.04 (3.84, 4.24) |  | 3.37 (3.19, 3.55) |  |
| 2014 | 2.39 (2.26, 2.54) |  | 2.21 (2.08, 2.35) |  | 3.94 (3.75, 4.14) |  | 3.54 (3.36, 3.72) |  |
| 2015 | 2.61 (2.47, 2.76) |  | 2.17 (2.04, 2.31) |  | 3.92 (3.73, 4.11) |  | 3.50 (3.33, 3.69) |  |
| 2016 | 2.53 (2.39, 2.68) |  | 2.25 (2.12, 2.38) |  | 3.93 (3.74, 4.12) |  | 3.72 (3.54, 3.91) |  |
| 2017 | 2.46 (2.32, 2.60) |  | 2.14 (2.01, 2.27) |  | 4.02 (3.83, 4.21) |  | 3.40 (3.23, 3.58) |  |
| 2018 | 2.51 (2.37, 2.65) |  | 2.15 (2.03, 2.28) |  | 4.07 (3.88, 4.26) |  | 3.56 (3.39, 3.74) |  |
| 2019 | 2.53 (2.40, 2.68) |  | 2.12 (2.00, 2.25) |  | 4.05 (3.87, 4.24) |  | 3.44 (3.28, 3.62) |  |
| 2020 | 2.55 (2.42, 2.69) |  | 2.17 (2.05, 2.30) |  | 4.20 (4.02, 4.39) |  | 3.69 (3.52, 3.87) |  |
| 2021 | 2.54 (2.40, 2.68) |  | 2.16 (2.03, 2.28) |  | 3.79 (3.62, 3.97) |  | 3.35 (3.19, 3.52) |  |
| AAIR, age-adjusted incidence rate; AAMR, age-adjusted mortality rate; AAPC, average annual percent change; CI, confidence interval. | | | | | | | | |
