# Supplementary material for: Evaluating short-term survivors of glioblastoma: A proposal based on SEER registry data
Source: Neurooncol Adv. 2025 Feb 9;7(1):vdaf036. doi: 10.1093/noajnl/vdaf036 (PMC12080546; doi:10.1093/noajnl/vdaf036)
Supplement: vdaf036_suppl_Supplementary_Table_S10 [file vdaf036_suppl_supplementary_table_s10.docx]

**Supplemental Table 10. Trends in age-adjusted incidence and mortality in glioblastoma by race/ethnicity**

|  | **Non-Hispanic White** | | | | **Non-Hispanic Black** | | | | **Non-Hispanic Asian and Pacific Islander** | | | | **Hispanic** | | | |
| --- | --- | --- | --- | --- | --- | --- | --- | --- | --- | --- | --- | --- | --- | --- | --- | --- |
|  | **Incidence** | | **Mortality** | | **Incidence** | | **Mortality** | | **Incidence** | | **Mortality** | | **Incidence** | | **Mortality** | |
| **Year** | **AAIR (95% CI))** | **AAPC** | **AAMR (95% CI)** | **AAPC** | **AAIR (95% CI))** | **AAPC** | **AAMR (95% CI)** | **AAPC** | **AAIR (95% CI))** | **AAPC** | **AAMR (95% CI)** | **AAPC** | **AAIR (95% CI))** | **AAPC** | **AAMR (95% CI)** | **AAPC** |
| 2000 | 3.53 (3.37, 3.70) | 0.42 (0.18, 0.68) | 1.49 (1.39, 1.60) | 1.07 (0.00, 2.25) | 1.86 (1.53, 2.25) | 0.56 (-0.01, 1.26) | 0.89 (0.66, 1.16) | 1.04 (0.22, 2.08) | 1.15 (0.88, 1.48) | 1.16 (0.37, 2.20) | 0.40 (0.24, 0.62) | 1.60 (0.58, 2.98) | 2.19 (1.84, 2.59) | 0.51  (0.09, 1.06) | 0.86 (0.63, 1.13) | 0.89 (0.04, 1.99) |
| 2001 | 3.45 (3.29, 3.61) |  | 2.66 (2.52, 2.81) |  | 1.76 (1.44, 2.13) |  | 1.52 (1.22, 1.88) |  | 1.39 (1.09, 1.75) |  | 0.91 (0.66, 1.21) |  | 2.46 (2.10, 2.86) |  | 1.79 (1.47, 2.14) |  |
| 2002 | 3.51 (3.35, 3.68) |  | 3.07 (2.92, 3.23) |  | 1.44 (1.15, 1.77) |  | 1.31 (1.03, 1.63) |  | 1.57 (1.26, 1.93) |  | 1.31 (1.03, 1.64) |  | 2.50 (2.15, 2.90) |  | 1.95 (1.63, 2.31) |  |
| 2003 | 3.67 (3.51, 3.84) |  | 3.24 (3.09, 3.40) |  | 1.87 (1.55, 2.25) |  | 1.28 (1.01, 1.59) |  | 1.53 (1.23, 1.89) |  | 1.23 (0.95, 1.55) |  | 2.61 (2.25, 3.02) |  | 2.13 (1.81, 2.50) |  |
| 2004 | 3.82 (3.65, 3.99) |  | 3.06 (2.91, 3.21) |  | 1.60 (1.30, 1.93) |  | 1.48 (1.20, 1.80) |  | 1.36 (1.08, 1.68) |  | 0.88 (0.66, 1.15) |  | 2.57 (2.22, 2.97) |  | 2.09 (1.76, 2.45) |  |
| 2005 | 3.80 (3.63, 3.97) |  | 3.37 (3.22, 3.53) |  | 1.72 (1.40, 2.08) |  | 1.51 (1.22, 1.84) |  | 1.49 (1.21, 1.82) |  | 1.21 (0.95, 1.51) |  | 2.61 (2.27, 2.99) |  | 2.12 (1.80, 2.48) |  |
| 2006 | 3.49 (3.33, 3.65) |  | 3.09 (2.94, 3.24) |  | 1.67 (1.37, 2.00) |  | 1.29 (1.03, 1.60) |  | 1.32 (1.06, 1.62) |  | 1.18 (0.93, 1.47) |  | 2.43 (2.11, 2.78) |  | 1.95 (1.65, 2.27) |  |
| 2007 | 3.83 (3.67, 4.00) |  | 3.27 (3.12, 3.42) |  | 1.75 (1.45, 2.08) |  | 1.28 (1.01, 1.58) |  | 1.62 (1.34, 1.95) |  | 1.03 (0.80, 1.30) |  | 2.47 (2.15, 2.82) |  | 1.73 (1.47, 2.03) |  |
| 2008 | 3.71 (3.55, 3.88) |  | 3.16 (3.02, 3.32) |  | 1.64 (1.35, 1.97) |  | 1.50 (1.22, 1.82) |  | 1.40 (1.14, 1.70) |  | 1.11 (0.87, 1.38) |  | 2.51 (2.20, 2.85) |  | 1.96 (1.69, 2.27) |  |
| 2009 | 3.79 (3.55, 3.88) |  | 3.33 (3.18, 3.49) |  | 1.97 (1.66, 2.33) |  | 1.34 (1.08, 1.63) |  | 1.20 (0.96, 1.47) |  | 0.96 (0.75, 1.21) |  | 2.12 (1.84, 2.43) |  | 2.04 (1.76, 2.35) |  |
| 2010 | 3.65 (3.49, 3.82) |  | 3.20 (3.06, 3.36) |  | 1.89 (1.59, 2.24) |  | 1.65 (1.37, 1.98) |  | 1.66 (1.38, 1.97) |  | 1.17 (0.94, 1.44) |  | 2.49 (2.20, 2.81) |  | 1.88 (1.62, 2.17) |  |
| 2011 | 3.71 (3.55, 3.88) |  | 3.25 (3.10, 3.40) |  | 1.93 (1.62, 2.27) |  | 1.49 (1.22, 1.79) |  | 1.59 (1.33, 1.89) |  | 1.27 (1.04, 1.55) |  | 2.27 (1.99, 2.57) |  | 1.94 (1.68, 2.23) |  |
| 2012 | 3.94 (3.77, 4.11) |  | 3.33 (3.18, 3.49) |  | 1.88 (1.59, 2.21) |  | 1.57 (1.30, 1.88) |  | 1.67 (1.41, 1.96) |  | 1.30 (1.07, 1.56) |  | 2.33 (2.05, 2.63) |  | 1.92 (1.67, 2.20) |  |
| 2013 | 3.86 (3.69, 4.02) |  | 3.30 (3.15, 3.46) |  | 1.92 (1.63, 2.24) |  | 1.30 (1.06, 1.57) |  | 1.71 (1.45, 2.01) |  | 1.35 (1.11, 1.61) |  | 2.47 (2.19, 2.77) |  | 1.93 (1.68, 2.20) |  |
| 2014 | 3.72 (3.56, 3.88) |  | 3.38 (3.23, 3.54) |  | 1.68 (1.42, 1.98) |  | 1.68 (1.41, 1.98) |  | 1.61 (1.35, 1.89) |  | 1.38 (1.14, 1.64) |  | 2.41 (2.14, 2.70) |  | 2.12 (1.86, 2.40) |  |
| 2015 | 3.85 (3.69, 4.02) |  | 3.36 (3.22, 3.52) |  | 1.79 (1.52, 2.10) |  | 1.62 (1.36, 1.91) |  | 1.52 (1.28, 1.79) |  | 1.41 (1.18, 1.67) |  | 2.75 (2.47, 3.06) |  | 2.06 (1.82, 2.33) |  |
| 2016 | 3.79 (3.63, 3.96) |  | 3.59 (3.44, 3.75) |  | 1.77 (1.49, 2.07) |  | 1.62 (1.36, 1.92) |  | 1.87 (1.61, 2.16) |  | 1.25 (1.04, 1.49) |  | 2.40 (2.14, 2.68) |  | 2.18 (1.93, 2.46) |  |
| 2017 | 3.79 (3.64, 3.96) |  | 3.32 (3.17, 3.47) |  | 1.96 (1.68, 2.27) |  | 1.44 (1.20, 1.72) |  | 1.46 (1.23, 1.71) |  | 1.28 (1.07, 1.52) |  | 2.62 (2.36, 2.90) |  | 2.06 (1.83, 2.32) |  |
| 2018 | 3.89 (3.73, 4.06) |  | 3.42 (3.27, 3.57) |  | 1.90 (1.63, 2.21) |  | 1.65 (1.40, 1.94) |  | 1.64 (1.41, 1.90) |  | 1.36 (1.15, 1.60) |  | 2.63 (2.38, 2.90) |  | 1.98 (1.76, 2.22) |  |
| 2019 | 3.88 (3.72, 4.05) |  | 3.38 (3.24, 3.53) |  | 1.87 (1.60, 2.16) |  | 1.71 (1.46, 1.99) |  | 1.68 (1.45, 1.95) |  | 1.19 (0.99, 1.41) |  | 2.79 (2.52, 3.07) |  | 1.98 (1.76, 2.22) |  |
| 2020 | 3.97 (3.81, 4.14) |  | 3.46 (3.31, 3.61) |  | 2.02 (1.75, 2.33) |  | 1.50 (1.26, 1.76) |  | 1.95 (1.70, 2.23) |  | 1.50 (1.29, 1.74) |  | 2.65 (2.40, 2.91) |  | 2.40 (2.16, 2.66) |  |
| 2021 | 3.77 (3.62, 3.93) |  | 3.29 (3.15, 3.44) |  | 1.80 (1.54, 2.09) |  | 1.49 (1.26, 1.75) |  | 1.53 (1.31, 1.77) |  | 1.29 (1.09, 1.52) |  | 2.64 (2.40, 2.91) |  | 2.14 (1.92, 2.38) |  |
| AAIR, age-adjusted incidence rate; AAMR, age-adjusted mortality rate; AAPC, average annual percent change; CI, confidence interval. | | | | | | | | | | | | | | | | |
