# Supplementary material for: Evaluating short-term survivors of glioblastoma: A proposal based on SEER registry data
Source: Neurooncol Adv. 2025 Feb 9;7(1):vdaf036. doi: 10.1093/noajnl/vdaf036 (PMC12080546; doi:10.1093/noajnl/vdaf036)
Supplement: vdaf036_suppl_Supplementary_Table_S11 [file vdaf036_suppl_supplementary_table_s11.docx]

**Supplemental Table 11. Total annual age-adjusted incidence rates and age-adjusted mortality rates by 5-year-age group**

|  | **All cases** | | | **Short-term survivors** | | | **Long-term survivors** | | |
| --- | --- | --- | --- | --- | --- | --- | --- | --- | --- |
| **Age groups (years)** | **AAIR (95% CI)** | **AAMR (95% CI)** | **MIR** | **AAIR (95% CI)** | **AAMR (95% CI)** | **MIR** | **AAIR (95% CI)** | **AAMR (95% CI)** | **MIR** |
| 00 years | 0.20 (0.15, 0.26) | 0.08 (0.0, 0.13) | 0.41 | 0.10 (0.06, 0.15) | 0.08 (0.05, 0.13) | 0.83 | 0.03 (0.02, 0.07) | 0.00 (0.00, 0.02) | 0.00 |
| 01-04 years | 0.08 (0.06, 0.09) | 0.03 (0.02, 0.05) | 0.45 | 0.02 (0.01, 0.03) | 0.02 (0.01, 0.03) | 0.84 | 0.01 (0.01, 0.02) | 0.00 (0.00, 0.00) | 0.00 |
| 05-09 years | 0.13 (0.11, 0.15) | 0.09 (0.08, 0.11) | 0.74 | 0.03 (0.02, 0.04) | 0.03 (0.02, 0.04) | 1.03 | 0.01 (0.00, 0.01) | 0.00 (0.00, 0.01) | 0.24 |
| 10-14 years | 0.16 (0.14, 0.19) | 0.12 (0.10, 0.14) | 0.73 | 0.03 (0.02, 0.04) | 0.03 (0.02, 0.04) | 1.00 | 0.02 (0.02, 0.03) | 0.01 (0.00, 0.01) | 0.23 |
| 15-19 years | 0.20 (0.18, 0.23) | 0.15 (0.13, 0.17) | 0.73 | 0.04 (0.03, 0.05) | 0.03 (0.02, 0.04) | 0.91 | 0.03 (0.02, 0.04) | 0.01 (0.01, 0.02) | 0.49 |
| 20-24 years | 0.26 (0.24, 0.29) | 0.16 (0.14, 0.19) | 0.62 | 0.04 (0.03, 0.04) | 0.03 (0.02, 0.04) | 1.00 | 0.06 (0.05, 0.08) | 0.02 (0.01, 0.03) | 0.29 |
| 25-29 years | 0.43 (0.39, 0.46) | 0.22 (0.20, 0.25) | 0.52 | 0.05 (0.04, 0.07) | 0.05 (0.03, 0.06) | 0.86 | 0.11 (0.09, 0.13) | 0.04 (0.03, 0.05) | 0.32 |
| 30-34 years | 0.60 (0.56, 0.64) | 0.33 (0.30, 0.36) | 0.55 | 0.09 (0.07, 0.11) | 0.08 (0.07, 0.10) | 0.92 | 0.14 (0.12, 0.16) | 0.06 (0.05, 0.07) | 0.42 |
| 35-39 years | 0.91 (0.86, 0.97) | 0.56 (0.52, 0.60) | 0.61 | 0.14 (0.12, 0.16) | 0.13 (0.11, 0.16) | 0.94 | 0.18 (0.16, 0.21) | 0.08 (0.06, 0.09) | 0.43 |
| 40-44 years | 1.56 (1.50, 1.63) | 1.05 (0.99, 1.11) | 0.67 | 0.30 (0.27, 0.33) | 0.27 (0.24, 0.30) | 0.90 | 0.22 (0.20, 0.25) | 0.11 (0.09, 0.13) | 0.48 |
| 45-49 years | 2.71 (2.62, 2.80) | 1.95 (1.88, 2.03) | 0.72 | 0.59 (0.55, 0.63) | 0.53 (0.49, 0.57) | 0.90 | 0.31 (0.28, 0.34) | 0.18 (0.16, 0.20) | 0.58 |
| 50-54 years | 4.49 (4.37, 4.61) | 3.37 (3.26, 3.47) | 0.75 | 1.17 (1.11, 1.23) | 1.03 (0.98, 1.09) | 0.88 | 0.45 (0.41, 0.49) | 0.26 (0.23, 0.29) | 0.58 |
| 55-59 years | 6.86 (6.70, 7.01) | 5.56 (5.42, 5.70) | 0.81 | 2.03 (1.95, 2.12) | 1.85 (1.77, 1.94) | 0.91 | 0.57 (0.53, 0.62) | 0.38 (0.35, 0.42) | 0.67 |
| 60-64 years | 9.32 (9.12, 9.52) | 8.00 (7.82, 8.19) | 0.86 | 3.39 (3.27, 3.51) | 3.10 (2.99, 3.22) | 0.92 | 0.60 (0.56, 0.66) | 0.51 (0.46, 0.56) | 0.84 |
| 65-69 years | 11.71 (11.46, 11.96) | 10.34 (10.11, 10.58) | 0.88 | 5.26 (5.09, 5.43) | 4.77 (4.61, 4.93) | 0.91 | 0.57 (0.51, 0.62) | 0.56 (0.51, 0.62) | 0.99 |
| 70-74 years | 14.36 (14.05, 14.68) | 13.06 (12.76, 13.36) | 0.91 | 7.72 (7.49, 7.95) | 7.13 (6.91, 7.36) | 0.92 | 0.41 (0.36, 0.47) | 0.51 (0.45, 0.57) | 1.25 |
| 75-79 years | 15.58 (15.21, 15.96) | 14.44 (14.08, 14.81) | 0.93 | 10.08 (9.78, 10.39) | 9.27 (8.98, 9.57) | 0.92 | 0.28 (0.23, 0.33) | 0.39 (0.33, 0.45) | 1.40 |
| 80-84 years | 14.30 (13.88, 14.73) | 13.74 (13.32, 14.16) | 0.96 | 11.00 (10.63, 11.38) | 10.29 (9.94, 10.66) | 0.94 | 0.13 (0.09, 0.18) | 0.23 (0.18, 0.29) | 1.75 |
| 85+ years | 9.09 (8.75, 9.43) | 8.87 (8.54, 9.21) | 0.98 | 7.75 (7.44, 8.07) | 7.35 (7.05, 7.67) | 0.95 | 0.04 (0.02, 0.07) | 0.09 (0.06, 0.13) | 2.36 |
| AAIR, age-adjusted incidence rate; AAMR, age-adjusted mortality rate; CI, confidence interval; MIR, mortality-to-indcidence ratio. | | | | | | | | | |
