# Supplementary material for: Evaluating short-term survivors of glioblastoma: A proposal based on SEER registry data
Source: Neurooncol Adv. 2025 Feb 9;7(1):vdaf036. doi: 10.1093/noajnl/vdaf036 (PMC12080546; doi:10.1093/noajnl/vdaf036)
Supplement: vdaf036_suppl_Supplementary_Table_S12 [file vdaf036_suppl_supplementary_table_s12.docx]

**Supplemental Table 12. Multivariate Fine-Gray’s analysis for glioblastoma regarding cases with other causes of death as “dead” rather than “censored”.**

|  | | **Short-term survivor** | | |  | **Intermediate-term survivor** | | |  | **Long-term survivor** | | |
| --- | --- | --- | --- | --- | --- | --- | --- | --- | --- | --- | --- | --- |
| **Characteristics** | | **SHR** | **95% CI** | ***p*-value** |  | **SHR** | **95% CI** | ***p*-value** |  | **SHR** | **95% CI** | ***p*-value** |
| Age at diagnosis | |  |  |  |  |  |  |  |  |  |  |  |
|  | 40-69 years | Reference |  |  |  | Reference |  |  |  | Reference |  |  |
|  | 0-14 years | 0.94 | 0.77-1.15 | 0.520 |  | 1.10 | 0.95-1.28 | 0.190 |  | 0.25 | 0.17-0.38 | <0.001 |
|  | 15-39 years | 1.15 | 1.04-1.28 | 0.008 |  | 0.69 | 0.65-0.72 | <0.001 |  | 0.56 | 0.50-0.62 | <0.001 |
|  | 70+ years | 1.03 | 1.01-1.06 | 0.013 |  | 1.38 | 1.33-1.43 | <0.001 |  | 1.41 | 1.26-1.58 | <0.001 |
| Sex | |  |  |  |  |  |  |  |  |  |  |  |
|  | Female | Reference |  |  |  | Reference |  |  |  | Reference |  |  |
|  | Male | 1.03 | 1.01-1.06 | 0.012 |  | 1.09 | 1.06-1.22 | <0.001 |  | 1.18 | 1.10-1.27 | <0.001 |
| Tumor location | |  |  |  |  |  |  |  |  |  |  |  |
|  | Cerebral cortex | Reference |  |  |  | Reference |  |  |  | Reference |  |  |
|  | Cerebellum | 1.10 | 0.96-1.27 | 0.160 |  | 1.05 | 0.83-1.33 | 0.690 |  | 0.88 | 0.60-1.27 | 0.480 |
|  | Midline | 1.19 | 1.01-1.40 | 0.039 |  | 1.32 | 1.10-1.58 | 0.003 |  | 1.05 | 0.69-1.61 | 0.810 |
| Race | |  |  |  |  |  |  |  |  |  |  |  |
|  | NHW | Reference |  |  |  | Reference |  |  |  | Reference |  |  |
|  | NHB | 0.93 | 0.88-0.99 | 0.015 |  | 0.97 | 0.92-1.03 | 0.300 |  | 1.09 | 0.93-1.27 | 0.280 |
|  | NHAPI | 0.89 | 0.84-0.95 | <0.001 |  | 0.87 | 0.82-0.92 | <0.001 |  | 1.06 | 0.92-1.22 | 0.430 |
|  | Hispanic | 0.95 | 0.91-0.99 | 0.010 |  | 0.94 | 0.91-0.98 | 0.006 |  | 0.90 | 0.80-1.01 | 0.060 |
| Treatment <3 weeks after diagnosis | |  |  |  |  |  |  |  |  |  |  |  |
|  | No | Reference |  |  |  | Reference |  |  |  | Reference |  |  |
|  | Yes | 1.12 | 1.08-1.16 | <0.001 |  | 1.02 | 0.98-1.07 | 0.370 |  | 1.07 | 0.97-1.19 | 0.180 |
|  | Extent of resection |  |  |  |  |  |  |  |  |  |  |  |
|  | Subtotal resection | Reference |  |  |  | Reference |  |  |  | Reference |  |  |
|  | Gross total resection | 0.93 | 0.90-0.95 | <0.001 |  | 0.92 | 0.90-0.95 | <0.001 |  | 0.97 | 0.90-1.05 | 0.510 |
| Radiation therapy | |  |  |  |  |  |  |  |  |  |  |  |
|  | No | Reference |  |  |  | Reference |  |  |  | Reference |  |  |
|  | Yes | 0.59 | 0.57-0.61 | <0.001 |  | 0.95 | 0.89-1.01 | 0.075 |  | 1.09 | 0.94-1.26 | 0.270 |
| Chemotherapy | |  |  |  |  |  |  |  |  |  |  |  |
|  | No | Reference |  |  |  | Reference |  |  |  | Reference |  |  |
|  | Yes | 0.75 | 0.73-0.78 | <0.001 |  | 0.70 | 0.67-0.73 | <0.001 |  | 1.09 | 0.97-1.24 | 0.160 |
