# Supplementary material for: Evaluating short-term survivors of glioblastoma: A proposal based on SEER registry data
Source: Neurooncol Adv. 2025 Feb 9;7(1):vdaf036. doi: 10.1093/noajnl/vdaf036 (PMC12080546; doi:10.1093/noajnl/vdaf036)
Supplement: vdaf036_suppl_Supplementary_Table_S13 [file vdaf036_suppl_supplementary_table_s13.docx]

**Supplemental Table 13. Annual age-adjusted mortality rates and ratios in decedents with GBM-specific death by sex and each age subgroup**

|  |  | **Short-term survivors** | | |  | **Long-term survivors** | | |
| --- | --- | --- | --- | --- | --- | --- | --- | --- |
| **Age groups (years)** | **Sex** | **AAMR (95% CI)** | **Rate ratio (95% CI)** | ***p* value** |  | **AAMR (95% CI)** | **Rate ratio (95% CI)** | ***p* value** |
| 0-14 | Male | 0.03 (0.02, 0.04) | reference | |  | 0.00 (0.00, 0.01) | reference | |
|  | Female | 0.03 (0.02, 0.04) | 0.88 (0.59, 1.29) | 0.545 |  | 0.00 (0.00, 0.01) | 1.33 (0.29, 6.70) | 0.927 |
| 15-39 | Male | 0.09 (0.08, 0.10) | reference | |  | 0.05 (0.05, 0.06) | reference | |
|  | Female | 0.05 (0.04, 0.06) | 0.54 (0.43, 0.66) | <0.001 |  | 0.03 (0.02, 0.04) | 0.56 (0.43, 0.73) | <0.001 |
| 40-69 | Male | 1.81 (1.77, 1.86) | reference | |  | 0.33 (0.31, 0.35) | reference | |
|  | Female | 1.16 (1.12, 1.19) | 0.64 (0.61, 0.66) | <0.001 |  | 0.23 (0.22, 0.25) | 0.70 (0.64, 0.76) | <0.001 |
| 70+ | Male | 10.71 (10.46, 10.96) | reference | |  | 0.43 (0.38, 0.48) | reference | |
|  | Female | 6.81 (6.64, 6.98) | 0.64 (0.61, 0.66) | <0.001 |  | 0.29 (0.25, 0.32) | 0.67 (0.56, 0.79) | <0.001 |
| AAMR, age-adjusted mortality rate; CI, confidence interval; GBM, glioblastoma. | | | | | | | | |
