# Supplementary material for: Evaluating short-term survivors of glioblastoma: A proposal based on SEER registry data
Source: Neurooncol Adv. 2025 Feb 9;7(1):vdaf036. doi: 10.1093/noajnl/vdaf036 (PMC12080546; doi:10.1093/noajnl/vdaf036)
Supplement: vdaf036_suppl_Supplementary_Table_S14 [file vdaf036_suppl_supplementary_table_s14.docx]

**Supplemental Table 14. Annual age-adjusted incidence rates and ratio in patients with GBM-specific death by sex and each age subgroups**

|  |  | **Short-term survivors** | | |  | **Long-term survivors** | | |
| --- | --- | --- | --- | --- | --- | --- | --- | --- |
| **Age groups (years)** | **Sex** | **AAIR (95% CI)** | **Rate ratio (95% CI)** | **p value** |  | **AAIR (95% CI)** | **Rate ratio (95% CI)** | **p value** |
| 0-14 | Male | 0.03 (0.03, 0.04) | reference | |  | 0.01 (0.00, 0.01) | reference | |
|  | Female | 0.03 (0.02, 0.04) | 0.87 (0.59, 1.27) | 0.498 |  | 0.01 (0.00, 0.01) | 1.46 (0.60, 3.68) | 0.472 |
| 15-39 | Male | 0.09 (0.08, 0.10) | reference | |  | 0.08 (0.07, 0.09) | reference | |
|  | Female | 0.05 (0.04, 0.06) | 0.54 (0.43, 0.66) | <0.001 |  | 0.05 (0.04, 0.06) | 0.61 (0.49, 0.75) | <0.001 |
| 40-69 | Male | 1.81 (1.81, 1.90) | reference | |  | 0.33 (0.31, 0.35) | reference | |
|  | Female | 1.19 (1.15, 1.22) | 0.64 (0.61, 0.66) | <0.001 |  | 0.36 (0.34, 0.38) | 0.71 (0.65, 0.78) | <0.001 |
| 70+ | Male | 10.46 (10.21, 10.71) | reference | |  | 0.19 (0.16, 0.23) | reference | |
|  | Female | 6.70 (6.54, 6.87) | 0.64 (0.62, 0.66) | <0.001 |  | 0.15 (0.12, 0.17) | 0.75 (0.59, 0.97) | 0.025 |
| AAIR, age-adjusted incidence rate; CI, confidence interval; GBM, glioblastoma. | | | | | | | | |
